# Curcumin (Ferrocene)-Functionalized Polyurethane Composite Dressing Integrated with Dopamine-Grafted Sodium Alginate: Synergistic ROS Modulation and Durable Tissue Adhesion

**DOI:** 10.3390/jfb17090473

**Published:** 2026-09-17

**Authors:** Jiacheng Yu, Chengming Wang, Xiue Ren, Huixia Wang, Yiqiang Huang, Changren Zhou

**Affiliations:** 1 School of Life Sciences, Zhuhai College of Science and Technology, Zhuhai 519040, China; 2 College of Chemistry, Jilin University, Changchun 130012, China

**Keywords:** curcumin, polyurethane, anti-inflammatory, wound healing, ROS scavenging

## Abstract

Elevated levels of reactive oxygen species (ROS) and persistent inflammatory responses represent principal impediments to efficacious wound healing, particularly in mechanically dynamic or infected wound environments. Consequently, multifunctional hydrogel-based dressings capable of integrating coordinated antioxidant, anti-inflammatory, and antimicrobial activities with robust wet-tissue adhesion are highly sought after, yet their development remains limited. Herein, we introduce a curcumin-functionalized polyurethane composite dressing (designated CPFSD), engineered through the incorporation of dopamine-grafted sodium alginate (SD) and ferrocene (Fc), which collectively confer synergistic ROS modulation alongside durable tissue adhesion. Mechanistically, curcumin provides intrinsic antioxidant and anti-inflammatory properties, while Fc facilitates reversible Fe^2+^/Fe^3+^ redox cycling, thereby augmenting ROS scavenging capacity; concurrently, catechol and hydroquinone moieties present on SD establish stable interfacial interactions with moist biological tissues. Comprehensive physicochemical characterization revealed that CPFSD effectively scavenged over 80% of both DPPH and hydroxyl (·OH) radicals, exhibited greater than 80% antibacterial efficacy against Escherichia coli and Staphylococcus aureus, and demonstrated markedly enhanced adhesive performance while preserving mechanical flexibility and cytocompatibility. In vitro assays further indicated that CPFSD significantly attenuated oxidative stress in L929 fibroblasts and RAW264.7 macrophages, accompanied by downregulation of pro-inflammatory cytokine expression. In a murine full-thickness excisional wound model, CPFSD facilitated accelerated epithelialization, angiogenesis, and wound contraction, achieving wound closure at day 11 without observable systemic toxicity. Collectively, these findings underscore that rational multicomponent design strategies can yield adhesion-capable wound dressings endowed with synergistic therapeutic functionalities suitable for addressing complex pathological wound conditions.

## 1. Introduction

Wound healing is a dynamically regulated biological process consisting of four overlapping phases: hemostasis, inflammation, proliferation, and remodeling [1]. Persistent reactive oxygen species (ROS) overaccumulation and uncontrolled inflammatory activation are major pathological obstacles that disrupt wound-site microenvironment homeostasis and hinder skin regeneration, frequently resulting in delayed or chronic wound healing [2,3,4]. Excess ROS at injury sites initiates a self-amplifying crosstalk with inflammatory signaling pathways, further driving cytokine over-secretion, prolonging the inflammatory phase, and elevating susceptibility to bacterial colonization [5,6,7,8]. Therefore, ideal wound dressings are expected to integrate effective ROS scavenging, anti-inflammatory regulation, antibacterial capability, and reliable wet-tissue adhesion to maintain stable dressing-tissue contact under mechanical motion [9,10,11].

Nevertheless, even when individual therapeutic effects such as ROS scavenging, anti-inflammation, or bacteriostasis are achieved, it remains difficult to break the self-sustaining ROS–inflammation positive-feedback loop within complex pathological wounds relying solely on these discrete therapeutic outputs [12,13,14]. Although numerous multifunctional hydrogel dressings have been reported by combining multiple active ingredients, most of them merely adopt simple physical mixing of functional components rather than constructing molecular-level synergistic regulatory pathways, which restricts their comprehensive therapeutic performance [15,16,17]. In addition to insufficient coordinated therapeutic effects, inadequate wet-tissue adhesion constitutes another prominent bottleneck for practical application. Many reported hydrogel dressings achieve adhesion primarily via weak physical adsorption or fragile hydrogen-bond interactions, which are vulnerable to failure under mechanical deformation of wound tissues. Mechanical movement of wound sites easily causes dressing detachment, destroying the moist regenerative microenvironment and inducing secondary friction-caused damage to nascent tissue, which in turn exacerbates local inflammation and delays repair [18,19,20].

Current mainstream strategies to endow hydrogels with ROS-scavenging capacity mainly include physical encapsulation of antioxidants or covalent grafting of active moieties onto polymer backbones [21,22]. Correspondingly, antibacterial functions are commonly realized by loading antibiotics, metal nanoparticles, or antimicrobial peptides [23,24,25]. However, direct incorporation of these functional additives often increases formulation complexity and may compromise material stability and cytocompatibility, making it difficult to simultaneously achieve antioxidant, anti-inflammatory, and antibacterial synergies for treating multifactorial wounds such as diabetic ulcers [26,27,28].

To address the above-mentioned dual limitations of insufficient synergistic therapy and poor wet-adhesive performance of existing wound dressings, we herein construct a curcumin-functionalized polyurethane composite dressing (CPFSD). Dopamine-grafted sodium alginate (SD) with abundant catechol groups and ferrocene (Fc) are introduced into the polyurethane matrix to build a multifunctional platform combining robust wet-tissue adhesion and coordinated therapeutic effects. Mechanistically, curcumin provides intrinsic antioxidant and anti-inflammatory activity, while ferrocene participates in reversible Fe^2+^/Fe^3+^ redox cycling to amplify ROS-scavenging capacity. The catechol groups derived from SD not only enable stable interfacial bonding with moist biological tissues, but also immobilize iron species dissociated from ferrocene to avoid excessive Fenton-derived ROS burst. Such multicomponent collaboration mitigates oxidative stress and inflammatory responses in wound microenvironments, while stable adhesion reduces dressing displacement and dressing-change frequency, preserving a moist pro-regenerative microenvironment and protecting fragile newly formed tissue. Collectively, this work proposes an integrated “synergistic-therapy-secure-adhesion” material strategy, providing a feasible design reference for advanced wound dressings oriented toward complex pathological wounds.

## 2. Materials and Methods

### 2.1. Experimental Materials

The following reagents were used: polyethylene glycol (PEG, Mn 2000), isophorone diisocyanate (IPDI), isosorbide (IS), pentaerythritol triacrylate (PETA, 96%), curcumin (AR, Cur), dibutyltin dilaurate (DBDTL, 95%), 1-(3-dimethylaminopropyl)-3-ethylcarbodiimide hydrochloride (EDC), N-hydroxysuccinimide (NHS), 2-(N-morpholino) ethanesulfonic acid (MES), dopamine hydrochloride (DA), and ferrocene were purchased from Macklin Biochemical Co., Ltd. (Shanghai, China).Sodium alginate with an M/G (D-mannuronate to L-guluronate) ratio of 1:1 (SA, 200 mPa·s, 1% mass fraction of the solution at 25 °C, molecular weight: 110,335 Da, AR) was purchased from Aladdin Chemistry Co., Ltd. (Shanghai, China).The mouse macrophage cell line RAW264.7 (catalog number: iCell-m047) and mouse fibroblast cell line L929 (catalog number: iCell-m003) were purchased from iCell Bioscience Inc. (Shanghai, China). DMEM medium was obtained from Gibco (Grand Island, NY, USA), and MEM medium was sourced from Cytiva (Marlborough, MA, USA). LPS, mouse TNF-α, IL-6 kit, and reactive oxygen species assay kit were purchased from Beyotime Biotechnology (Shanghai, China). Penicillin–streptomycin 100× (double antibody) and trypsin were acquired from White Shark (Hefei, China). Fetal bovine serum (FBS) and PBS were obtained from Procell Life Science & Technology Co., Ltd. (Wuhan, China). CCK8 was purchased from Invigentech Co., Ltd. (Nanjing, China), while GSH-PX, MDA, NO, MDA-PX, and ATP kits were sourced from Nanjing Jiancheng Bioengineering Institute (Nanjing, China). The H_2_O_2_ assay kit was obtained from Solarbio Science & Technology Co., Ltd. (Beijing, China), and the mouse IL-1β kit was provided by Jingmei (Yancheng, China). Isoflurane was sourced from RWD Life Science Co., Ltd. (Shenzhen, China), saline from Shandong Qidu Pharmaceuticals (Zibo, China), and tribromoethanol from Sigma-Aldrich (St. Louis, MO, USA). 3M™ Tegaderm™ was used as the control dressing, while the wound dressing was Yunnan Baiyao Waterproof Band-Aids (Yunnan Baiyao Group Co., Ltd., Kunming, China).

### 2.2. Preparation of Curcumin-Based Polyurethane (CPU)

Before beginning the reaction, nitrogen gas was purged through the three-neck flask to displace air inside the reactor. Once the temperature reached 70 °C, polyethylene glycol (PEG2000, 50.4 g) was added dropwise, after which the mixture was further heated to 90 °C. At this point, isophorone diisocyanate (IPDI, 11 mL) and the catalyst dibutyltin dilaurate (DBTDL) were introduced. Isocyanate groups reacted with hydroxyl groups under vigorous stirring (400 rpm) for 1.5 h to form an isocyanate-terminated polyurethane prepolymer. Subsequently, curcumin (Cur, 2 g) was completely dissolved in acetone and homogenized with isosorbide (IS, 2 g). The resulting homogeneous solution was added to the three-necked flask. The chain-extension reaction between isocyanate-terminated polyurethane prepolymer and hydroxyl groups of chain extenders proceeded under stirring at 400 rpm. The reaction was carried out at 90 °C under a nitrogen atmosphere for 4 h. Then, 12 mL of pentaerythritol triacrylate was added dropwise to the reaction mixture. The reaction was allowed to proceed for another 3.5 h, and the temperature was reduced to 70 °C to complete the reaction. Residual solvents were removed via vacuum distillation at 60 °C (5 kPa), yielding the final CPU product.

### 2.3. Preparation of Polydopamine-Alginate Composite (SD)

The solution with a fixed concentration of SA (0.5%; *w*/*v*%) was obtained by dissolving SA in deionized water at room temperature. When the SA powders were completely dissolved, the EDC/NHS (the ratio of EDC to NHS monomer was 1:1) was added to the SA solution with a molar ratio of EDC to SA repeating unit of 1:2.5 to activate the carboxyl groups of SA. MES (a buffer agent, 29 mM) was added, and the blending solution above was stirred for about 10 min to form a uniform solution. Finally, the pH of the uniform blending solution was adjusted to 5.5. After 2 h, DA was added to activate the SA solution with a mass ratio of DA to SA of 1:10, and the resulting solution was stirred at room temperature for 12 h. To remove the unreacted coupling reagents and micro-molecules, the obtained solution was dialyzed with a dialysis bag (molecular weight cut-off = 3500 Da) for 72 h, and deionized water was replaced every 3 h.

### 2.4. Preparation of CPFSD and PU-Fe

Curcumin-based polyurethane (CPU) was first dispersed in ethanol to prepare a stable precursor dispersion with a fixed solid concentration of 150 mg/mL, and 1% camphorquinone was incorporated as a photoinitiator prior to casting into a PTFE mold. A fixed volume of 8 mL precursor dispersion was poured into the PTFE mold with inner dimensions of 8 cm × 8 cm (casting area = 64 cm^2^) for thickness normalization. After degassing at 4 °C for 2 h, the mixture was UV-cured for 30 min (UV wavelength: 365 nm, light intensity: 10 mW·cm^−2^) and post-cured at room temperature for 48 h to afford CPU films. After cross-linking, films were further dried under well-ventilated ambient conditions to remove residual ethanol. The thickness of each film was measured at five random positions by a micrometer, and the film thickness was controlled at 120 ± 10 μm. For CPFSD1–CPFSD4 composite dressings, ferrocene content was fixed at 1 wt%, while SD mass fraction was varied from 10 wt% to 40 wt% to obtain different hydrophilic composite films. All CPFSD samples adopted identical casting volume, mold, degassing, UV-curing, and post-drying procedures to guarantee consistent film geometry across groups. The synthesis method of PU-Fe was similar to that of CPU. Briefly, polyethylene glycol (PEG2000) was reacted with isophorone diisocyanate (IPDI) at 90 °C, with dibutyltin dilaurate (DBTDL) added dropwise as the catalyst. The reaction was carried out at 400 rpm for 1.5 h, followed by the addition of isosorbide (IS) for a further reaction of 4 h. Subsequently, 12 mL of pentaerythritol triacrylate was added, and the mixture was reacted at 70 °C for 3.5 h. Thereafter, the residual solvent was removed by vacuum distillation at 60 °C under a pressure of 5 kPa to obtain the PU product. Then an equal dosage of ferrocene and camphorquinone was added to the mixture, and PU-Fe was finally obtained via UV curing. The detailed weight-percent compositional information for PU-Fe, CPU, and CPFSD-series composite dressings is summarized in Table S1 (Supporting Information).

### 2.5. Characterization

Characterization experiments include FTIR, microscopic morphology (SEM), X-ray photoelectron spectroscopy (XPS), X-ray diffraction (XRD), UV-Vis spectroscopy (UV), and ^1^H-NMR characterization. Physical properties include swelling rate, water absorption, contact angle measurements, water vapor transmission rate, ferrocene-induced ROS generation experiment, thermal stability, drug release properties, and degradation properties. Detailed information can be found in the Supporting Information Sections S1.1 and S1.2.

### 2.6. Mechanical Properties and Adhesive Properties

Tensile mechanical performance of hydrogel samples was measured by a universal mechanical testing machine (Gotech Testing Machines (Dongguan) Co., Ltd., Dongguan, China). The rectangular specimens (20 mm × 40 mm × 1 mm) were stretched at a constant rate of 50 mm·min^−1^ under ambient conditions. Wet-tissue adhesion behavior was characterized via lap-shear test and peel-strength measurement using fresh porcine skin as biological substrate. For the lap-shear assay, CPFSD samples were sandwiched between two porcine skin sheets with a bonding area of 20 mm × 20 mm, and the test was performed at 50 mm·min^−1^ following reported methods. The peel test was carried out according to ASTM F2258 standard at 50 mm·min^−1^ [19]. Adhesion toward non-biological substrates including glass, wood, rubber, and metal was also assessed. Each test was repeated at least three times. Calculation formula, reference, and additional photographs are provided in Supporting Information Sections S1.3 and S1.4.

### 2.7. In Vitro Coagulation Properties

The in vitro pro-coagulation capacity was evaluated by blood clotting index (BCI) and clotting-time assay using fresh rabbit whole blood. Briefly, 500 μL of pre-warmed whole blood was dropped onto pre-weighed hydrogel samples and incubated at 37 °C for 5 min. After incubation, 10 mL of deionized water was slowly added, and the absorbance of the supernatant at 540 nm was recorded to calculate BCI values. Supplementary raw data are available in Supporting Information Section S1.5.

### 2.8. In Vitro Biological Evaluation

The antioxidant capacity of hydrogels was determined by DPPH, hydroxyl radical, T-AOC, and superoxide-anion scavenging assays. Briefly, 0.1 g hydrogel samples were extracted with 5 mL 80% methanol in an ice-water bath for 30 min, followed by centrifugation at 12,000 rpm for 10 min to collect the supernatant for subsequent absorbance detection.

Antibacterial tests were carried out against *Staphylococcus aureus*, *Escherichia coli*, *Pseudomonas aeruginosa*, and *Staphylococcus epidermidis*. A bacterial viability assay was performed in LB medium with an initial bacterial concentration of 1 × 10^6^ CFU·mL^−1^ and incubated at 37 °C, 100 rpm for 24 h; bacterial growth was monitored via OD_600_. The agar-diffusion inhibition-zone test was also conducted using 5 mm-diameter hydrogel discs.

For cytocompatibility assessment, L929 fibroblasts and RAW264.7 macrophages were used. For the CCK-8 assay, cells were seeded at 100 μL per well in 96-well plates. After pre-culture, cells were treated with hydrogel extracts at gradient concentrations of 10, 30, 60, and 120 μg·mL^−1^ for 24 h. LIVE/DEAD fluorescence staining was performed at a cell density of 5000 cells·mL^−1^ with the same extract concentrations, and cells were cultured for 48 h.

For inflammatory stimulation experiments in six-well plates, L929 and RAW264.7 cells were seeded at 1 × 10^6^ cells per well and cultured overnight. Cells were pretreated with hydrogel extracts (30 μg·mL^−1^) for 1 h, then stimulated with LPS (10 μg·mL^−1^ for L929; 200 ng·mL^−1^ for RAW264.7) for an additional 24 h. Intracellular ROS was visualized by DCFH-DA staining under laser scanning confocal microscopy. The levels of intracellular NO, MDA, GSH, ATP, and inflammatory cytokines (TNF-α, IL-6, IL-1β) were quantified by corresponding assay kits. Hemocompatibility was evaluated using a red blood cell hemolysis test.

Calculation formulas for radical-scavenging efficiency, bacterial viability, ROS inhibition, and the Bliss independence synergistic model are summarized in Supporting Information Section S1.6. Additional experimental diagrams and original data are also provided in Supporting Information Section S1.6.

### 2.9. Animal Experiments

The efficacy of the dressing in treating full-thickness skin wounds in mice was evaluated using an 11 full-thickness skin wound model. All animal procedures were conducted following the National Research Council’s Guide for the Care and Use of Laboratory Animals (8th edition, NIH Publication, 2011). The levels of inflammatory factors such as MDA, GSH, ATP, TNF-α, IL-1β, and IL-6 during the wound healing process were measured using specific ELISA kits. Histological staining and immunohistochemical analysis were performed to evaluate epidermal regeneration, collagen deposition, inflammation, and angiogenesis in the wound tissue. Detailed information can be found in Supporting Information Section S1.7.

### 2.10. Statistical Analysis

All quantitative in vitro experiments were performed in triplicate or more independent biological replicates, and each group contained at least five biological replicates for in vivo animal experiments. Statistical analysis was performed using GraphPad Prism 9.5.0 and Origin 2018 software. Data are expressed as the mean ± SD. Student’s *t*-test was adopted for statistical comparison between two groups. For comparison among three or more groups, one-way analysis of variance (ANOVA) was performed, followed by Tukey’s multiple-comparison post-hoc test for multiple-comparison correction to reduce type-I error. Statistical significance was defined as *p* < 0.05 (0.01 < * *p* ≤ 0.05, ** *p* ≤ 0.01, *** *p* < 0.001, **** *p* < 0.0001).

## 3. Results

### 3.1. Preparation and Characterization of CPFSD

To elucidate the fabrication strategy and underlying mechanism of the multifunctional wound dressing, CPFSD was rationally designed by integrating curcumin-based polyurethane (CPU), ferrocene (Fc), and dopamine-grafted sodium alginate (SD) into a synergistic composite system.

**Figure 1:**
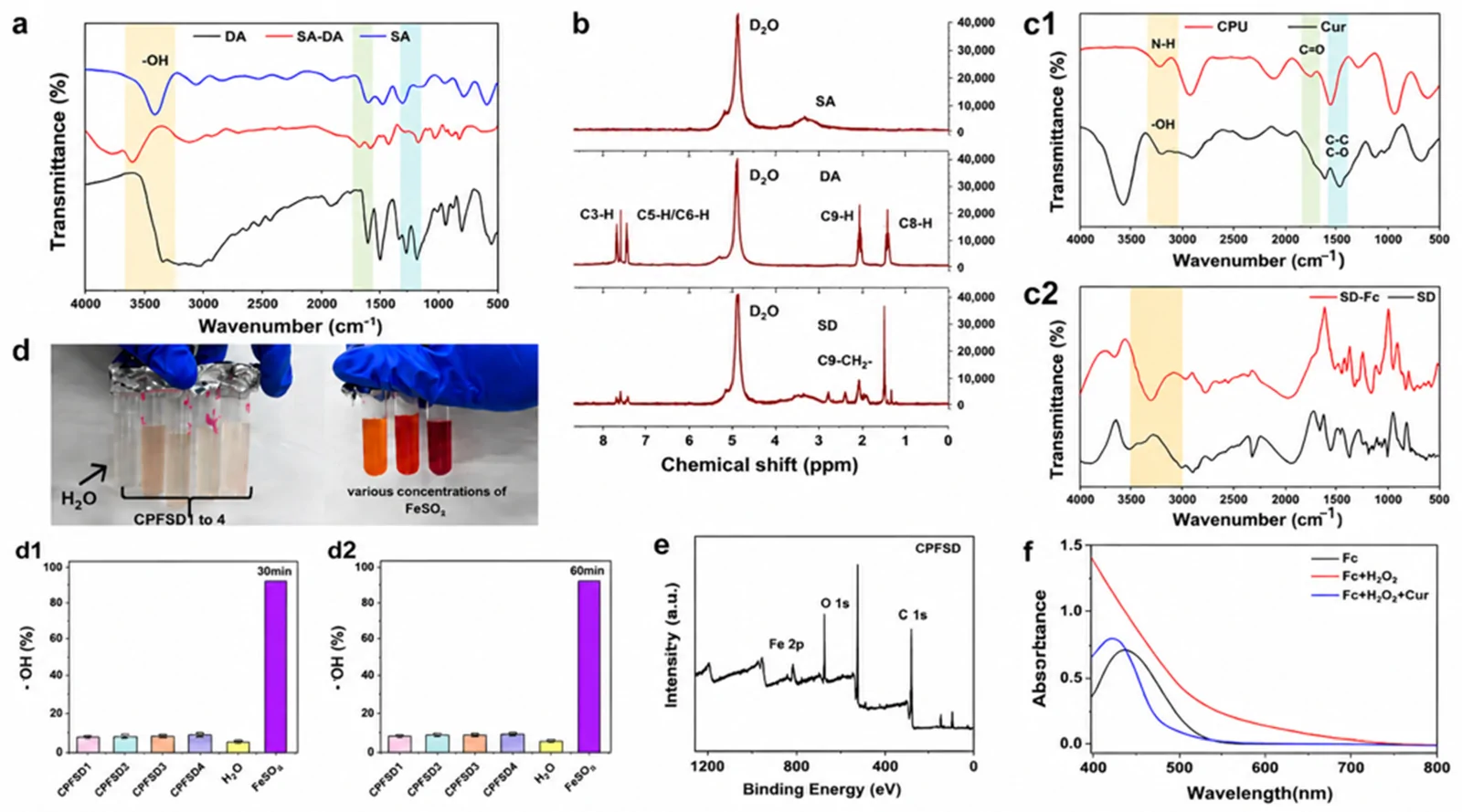
Characterization and microstructures of CPFSD dressings. (**a**) FTIR spectra of DA, SA and SD; (**b**) ^1^H NMR Spectra of SD, SA and DA; (**c1**) FTIR spectrum of CPU and Cur; (**c2**) FTIR spectrum of SD and Fc; (**d**) Visual phenomenon showing promoting effects of water, CPFSD and FeSO_4_ on hydroxyl radicals over a short time; (**d1**) Statistical results at 30 min; (**d2**) Statistical results at 60 min; (**e**) XPS spectra of CPFSD; (**f**) UV-Vis absorption spectra of CPU, CPFSD1, CPFSD2, CPFSD3 and CPFSD4.

First, the successful grafting of dopamine (DA) onto sodium alginate (SA) was verified to establish the functional basis of SD. As shown in the FTIR spectra (Figure 1a), SD exhibited characteristic peaks at 1616 cm^−1^ (C=C stretching) and 1287 cm^−1^ (C–O stretching of catechol groups) derived from DA. Meanwhile, the asymmetric and symmetric carboxylate stretching vibrations at 1617 cm^−1^ and 1417 cm^−1^, as well as the polysaccharide backbone C–O–C peak at 1033 cm^−1^, were well preserved in SD compared with SA. A broad O–H band at 3433 cm^−1^ and a C=O band at 1734 cm^−1^ (from oxidized aldehyde groups) remained unchanged after grafting, indicating retention of reactive moieties. Notably, new peaks at 1524 cm^−1^ and 1288 cm^−1^ corresponding to amide II and amide III vibrations confirmed the formation of amide bonds between DA and SA. The red-shifted O–H stretching band also suggested strengthened intramolecular hydrogen bonding after DA incorporation [29].

^1^H NMR analysis (Figure 1b) provided consistent structural evidence. SA showed typical resonances of guluronic (G) and mannuronic (M) units, while DA displayed aromatic signals (6.5–6.8 ppm) and aliphatic methylene signals (2.6–2.7 ppm). After grafting, SD retained all backbone signals of SA and introduced additional peaks assigned to catechol groups. Together, these results confirmed that DA was covalently grafted onto SA with intact catechol structure, which is critical for hydrophilicity, tissue adhesion, and ROS-scavenging properties of the final dressing [30,31].

Next, FTIR spectroscopy was employed to characterize polyurethane formation and curcumin integration in CPU with varied SD contents (Figure 1(c1,c2)). The characteristic O–H stretching peak of curcumin at ~3500 cm^−1^ disappeared in CPU, indicating that phenolic hydroxyl groups were consumed during urethane linkage formation. Concurrently, new peaks at 3232 cm^−1^ (N–H stretching) and 1766 cm^−1^ (C=O stretching) verified the successful synthesis of urethane bonds. Additional peaks at 709 cm^−1^ (aromatic C–H out-of-plane bending) and 1496 cm^−1^ (C=C and C=O vibrations) further confirmed the incorporation of curcumin into the polyurethane network.

As a redox-active component, the stabilization of Fc in the matrix was then investigated. As presented in Figure 1(c2), coordination between Fc and SD narrowed and weakened the broad O–H stretching band (3200–3600 cm^−1^), implying that catechol oxygen atoms coordinated with iron ions and weakened hydrogen-bonding interactions [32]. Such coordination was expected to immobilize Fc within the polymer matrix, preventing burst release and excessive ROS generation. To validate this hypothesis, ROS production was systematically tested (Figure 1d and Figure S1). The results demonstrated that CPFSD did not induce excessive ROS accumulation at 30, 60, 120 min, and 6 h.

X-ray photoelectron spectroscopy (XPS) was performed to analyze surface chemical compositions. The survey spectrum of CPFSD (Figure 1e) displayed strong signals at 285 eV (C 1s), 532 eV (O 1s), and 400 eV (N 1s), consistent with the elemental composition of the polyurethane framework. High-resolution C 1s spectra (Figure S2) were deconvoluted into three peaks at 284.78 eV (C–C/C–H), 286.72 eV (C–O–C/C–N), and 289.28 eV (C=O). The O 1s spectrum exhibited two peaks at 532.31 eV and 533.71 eV assigned to carbonyl and ether oxygen, respectively, and a single N 1s peak at 400.09 eV confirmed the presence of urethane nitrogen (–NH–C=O–).

The cross-sectional morphology of CPFSD was observed to evaluate the effect of SD content on microstructure (Figure S3). With increasing SD content, surface roughness increased noticeably, likely due to phase separation caused by the hydrophilic–hydrophobic contrast between sodium alginate and polyurethane [33]. Compared with the smooth surface of CPU, the grooved structure of CPFSD facilitated fluid absorption, gas permeation, and nutrient exchange [34].

UV–Vis spectroscopy further corroborated the successful integration of functional compositions (Figure S4). A distinct peak at 280 nm verified DA incorporation in SD, while the peak at 420 nm corresponded to the phenolic structure of curcumin. A weak peak near 320 nm was attributed to structural modification of curcumin during polymerization.

Finally, the reversible Fe^2+^/Fe^3+^ redox cycle responsible for ROS regulation was investigated (Figure 1f). Pure Fc showed a characteristic absorption peak at 440 nm (Fe^2+^). After treatment with H_2_O_2_, this peak vanished, and a broad band appeared, indicating oxidation to ferrocenium. Upon addition of curcumin, the 440 nm peak was restored, and the broad background absorbance decreased, demonstrating that curcumin reduced Fe^3+^ back to Fe^2+^. This reversible redox cycle confirmed that curcumin effectively mediated the Fe^2+^/Fe^3+^ switch, providing an explanation for the ROS-scavenging ability of CPFSD [35].

Notably, ferrous ions can trigger the Fenton reaction, producing toxic hydroxyl radicals that may aggravate oxidative tissue damage [36]. However, the CPFSD composite system suppresses excessive Fenton-derived ROS production through multiple synergistic mechanisms. First, ferrous/ferric ions dissociated from ferrocene are immobilized via coordination with catechol moieties of SD. This restricts the free diffusion of iron ions within the matrix and prevents high local concentrations of free Fe^2+^ that would otherwise drive vigorous Fenton reactions. Second, curcumin plays a dual role: it can reduce Fe^3+^ back to Fe^2+^, yet it also functions as an efficient radical scavenger to instantaneously quench hydroxyl radicals generated from minor Fenton side-reactions. Importantly, iron-ion immobilization by SD keeps the concentration of free Fe^2+^ at a low level, so that Fe^2+^ regeneration by curcumin does not initiate massive Fenton activity. Third, as shown in Figure 1d, the time-dependent ROS measurements confirmed that CPFSD did not cause excessive hydroxyl-radical accumulation from 30 min to 6 h of incubation. By comparison, the free FeSO_4_ positive control induced a sharp ROS burst under identical conditions. Collectively, SD-mediated immobilization of iron species combined with the radical-scavenging capacity of curcumin mitigates potential Fenton-associated risks. As a result, the CPFSD system favors ROS scavenging over the hazardous generation of hydroxyl radicals.

### 3.2. Physicochemical Properties of CPFSD

In this set of swelling experiments, the formulation of curcumin-based polyurethane (CPU) matrix and curcumin loading were fixed, while only the SD content was adjusted as the single experimental variable. It should be noted that both polyurethane network structure and curcumin content can modulate the swelling performance of composite hydrogels. Herein, the CPU matrix and curcumin fraction were pre-screened and optimized in our preliminary trials to balance mechanical properties and biological activity. By keeping the CPU-curcumin system constant, we could exclude interference originating from matrix variation and specifically investigate the influence of SD incorporation on the swelling characteristics of CPFSD dressings.

**Figure 2:**
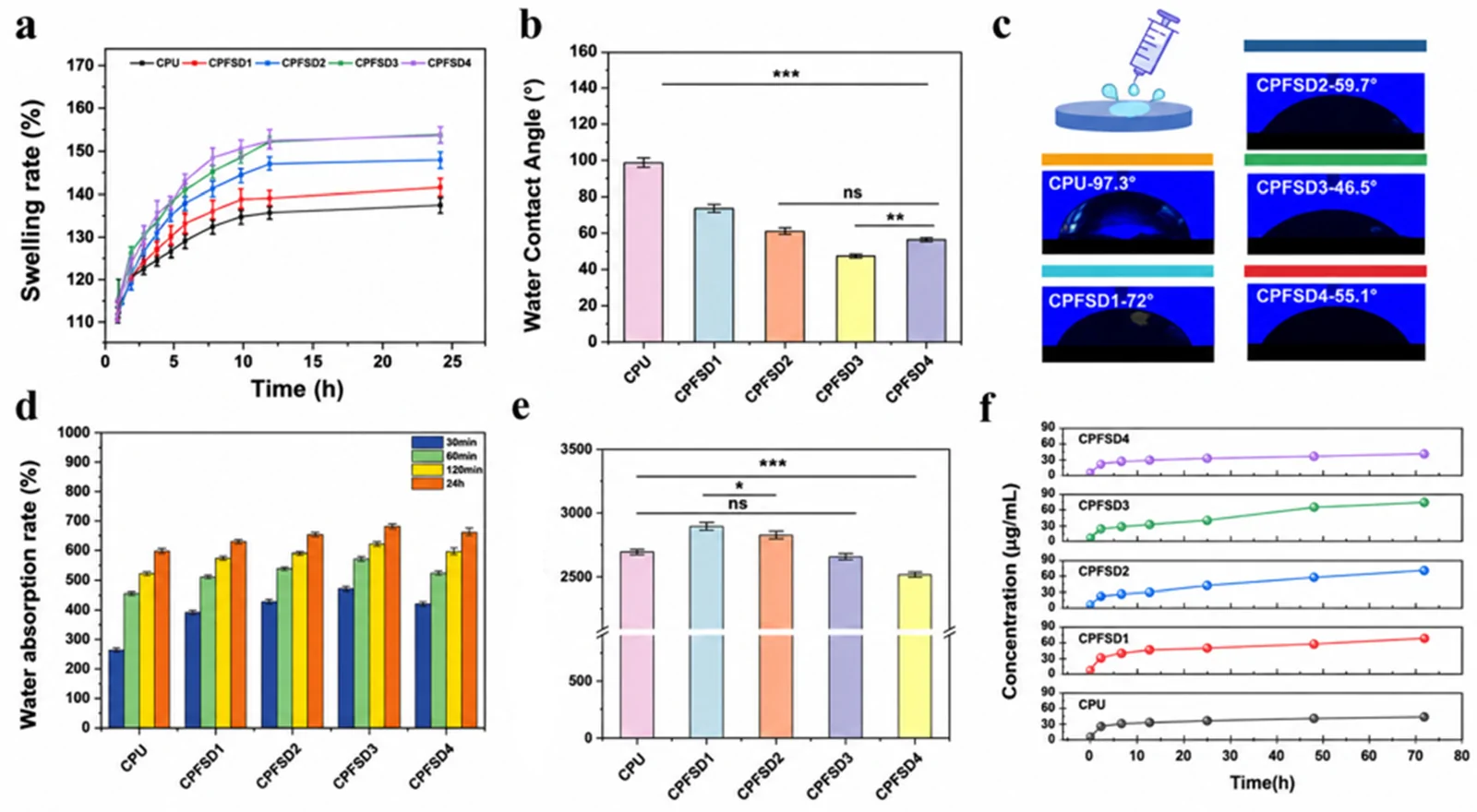
(**a**) Swelling rates of dressings with varying SD contents; (**b**,**c**) Water contact angles of dressings with varying SD contents; (**d**) Water absorption rates of dressings; (**e**) Water vapor transmission rates (WVTR; 37 °C; 60% RH); (**f**) Cumulative curcumin release profiles (Data are expressed as mean ± standard deviation, *n* = 3. ns: not significant, * *p* ≤ 0.05, ** *p* ≤ 0.01, *** *p* < 0.001).

Absorption and swelling properties are critical for wound dressings, as they regulate exudate handling, hemostasis, and tissue integration to accelerate wound repair [37]. Accordingly, the swelling behavior of dressings with varying SD loadings was systematically investigated. As illustrated in Figure 2a, all samples reached swelling equilibrium within 24 h, exhibiting rapid initial water uptake followed by a gradual slowdown until stabilization. The equilibrium swelling ratio rose from 138% for CPU (0% SD) to 154% for CPFSD3 (30% SD), then slightly decreased to 153% for CPFSD4 (40% SD), with only minor variations across groups. This trend can be explained by the molecular features of SD: dopamine grafting introduced plentiful hydrophilic polar groups (–COOH, –OH, –NH_2_), strengthening hydrogen-bonded water retention [38]. However, excessive SD loading (40%) caused intermolecular aggregation that partially shielded these hydrophilic sites, limiting further swelling improvement. Notably, such moderate swelling is favorable for wound management, as it preserves tight dressing–tissue contact, reduces peri-wound maceration, and maintains mechanical stability and durable adhesion for localized treatment [39].

To reveal the surface wettability underlying the swelling response, water contact angle (WCA) measurements were performed (Figure 2b). Incorporation of SD significantly improved the hydrophilicity and fluid-handling capacity of CPU dressings. With increasing SD content, the WCA sharply decreased from 97.3° (CPU, 0% SD) and reached the minimum value at CPFSD3. Further raising SD content to 40 wt% (CPFSD4) caused a slight rise in WCA, which agreed well with the swelling tendency. The enhanced wettability was attributed to two factors: the abundant hydrophilic groups introduced by dopamine-grafted SD, and the micro/nano-scale surface roughness generated by ferrocene-catalyzed oxidative cross-linking of catechol groups on SD chains. The slight WCA increase observed for CPFSD4 likely resulted from surface aggregation masking hydrophilic moieties, consistent with the swelling data [40].

In addition to surface wettability, water absorption capacity is a key index of exudate management performance. As shown in Figure 2d, the water absorption rate rose with SD concentration up to 30 wt% (CPFSD3) and decreased slightly at 40 wt% (CPFSD4), consistent with lower WCAs and improved hydrophilicity. This enhancement was driven by hydrophilic functional groups in SD that promoted hydrogen bonding with water molecules. All formulations achieved strong fluid uptake within 24 h, supporting efficient exudate removal and maintaining a moist microenvironment conducive to healing.

Water vapor transmission rate (WVTR) is essential for balancing moisture at the wound interface. All dressings displayed desirable WVTR values, yet permeability declined gradually with rising SD content (Figure 2e). This reduction was attributed to SD aggregation: higher loadings caused molecular stacking that partially blocked internal water vapor diffusion pathways, lowering WVTR [41]. These results confirm SD loading as a core factor controlling dressing performance, in line with trends in swelling, wettability, and water absorption.

To assess release behavior, a standard curcumin calibration curve was established (Figure 2f). Under simulated slightly acidic wound conditions (pH 6.5 and 7.4), SD incorporation promoted curcumin release, owing to improved hydrophilicity and water penetration that accelerated curcumin dissolution (Figure S5) [42]. At 40% SD, curcumin release decreased slightly, matching swelling and absorption trends and verifying the regulatory role of SD content. All dressings achieved cumulative curcumin release up to 45.67 μg/mL at pH 7.4 and 43.62 μg/mL at pH 6.5 within 24 h, demonstrating favorable sustained-release characteristics suitable for long-term wound care.

Collectively, these results demonstrate that dopamine-grafted sodium alginate (SD) effectively tailors the physicochemical properties of the wound dressings. These optimized characteristics establish a solid basis for the subsequent hemostatic, antibacterial, and tissue-repairing biological functions of the dressings.

### 3.3. Mechanical Properties of CPFSD

Given the dynamic mechanical environment of wounds, especially at mobile sites, suitable mechanical properties are essential for dressing durability and conformity to surrounding tissue. To clarify how SD concentration affects the mechanical and adhesive behavior of the films, mechanical tests were performed on CPFSD dressings with different SD loadings.

**Figure 3:**
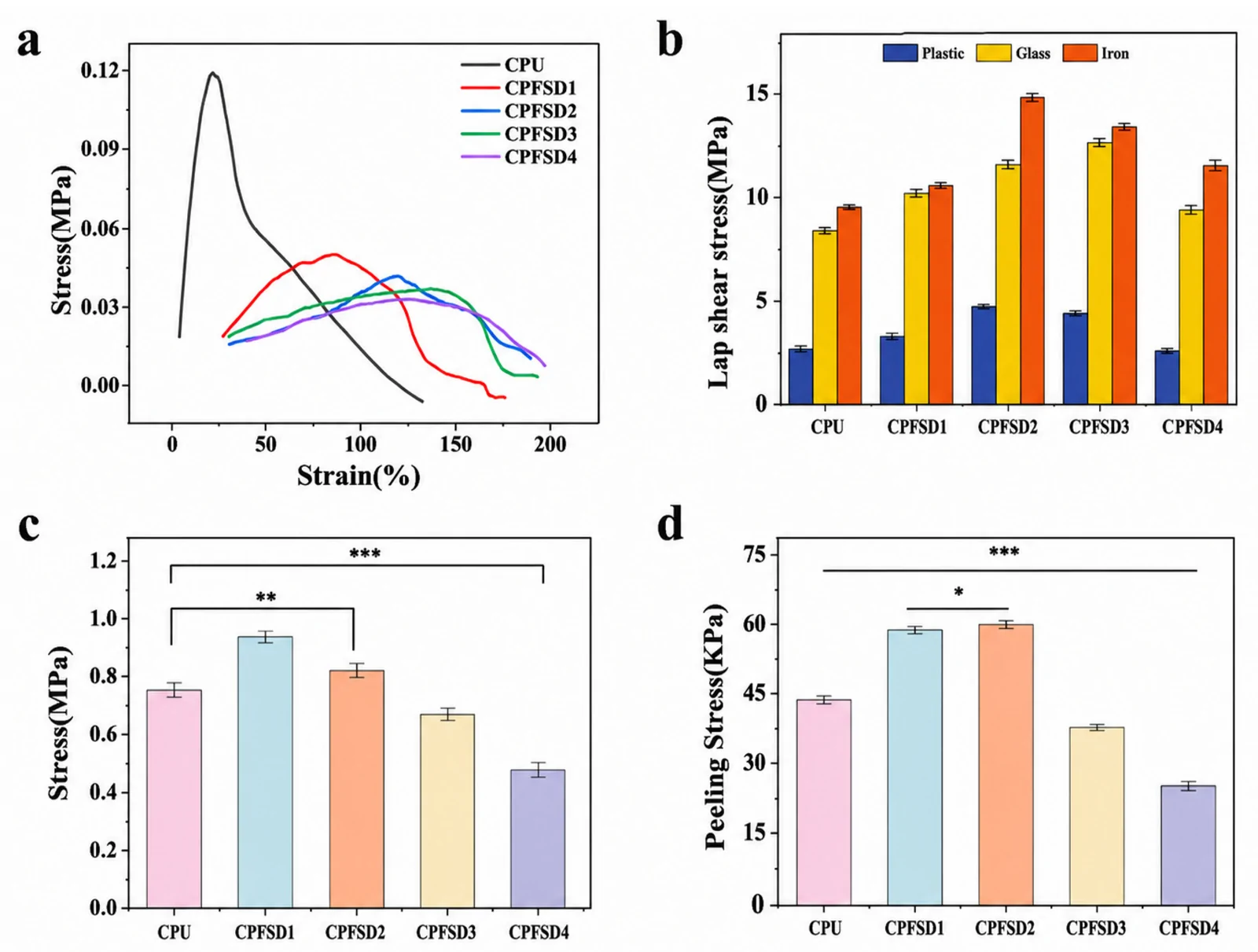
(**a**) Stress–strain curves; (**b**) Shear strength on plastic, glass, and metal; (**c**) Tensile adhesion strength on porcine skin; (**d**) Peel strength on porcine skin (Data are expressed as mean ± standard deviation, *n* = 3; * *p* ≤ 0.05, ** *p* ≤ 0.01, *** *p* < 0.001).

Flexibility is important for wound dressings because it allows them to adapt to complex body movements. The tensile stress–strain results show that SD incorporation increased the flexibility of CPFSD dressings but reduced their mechanical strength to some extent. In addition, adhesion to plastic, glass, and metal surfaces was evaluated to assess the effect of SD loading on interfacial bonding (Figure 3a). The shear strengths on plastic were 2.65 ± 0.17, 3.28 ± 0.16, 4.70 ± 0.07, 4.38 ± 0.12, and 2.56 ± 0.07 MPa for CPU, CPFSD1, CPFSD2, CPFSD3, and CPFSD4, respectively. On glass, the corresponding values were 8.35 ± 0.16, 10.18 ± 0.16, 11.52 ± 0.15, 12.60 ± 0.16, and 9.34 ± 0.20 MPa. On metal, the shear strengths were 9.50 ± 0.10, 10.50 ± 0.13, 14.50 ± 0.13, 14.80 ± 0.18, and 13.36 ± 0.15 MPa, respectively (Figure 3b).

Ideal bioadhesive materials are important for promoting biointegration and supporting tissue regeneration [43]. To further evaluate the tissue adhesion of CPU dressings with different SD concentrations, shear and peel tests were conducted using pig skin as a model tissue surface (Figure 3c,d). Previous studies have shown that catechol groups can significantly improve hydrogel adhesion to biological tissues [44]. In the shear test, CPFSD1 showed the highest adhesion strength, followed by CPFSD2 and CPU, whereas CPFSD3 and CPFSD4 showed lower adhesion than CPU. Consistent with this trend, the peel test gave adhesive strengths of 44.13 ± 0.70, 58.77 ± 0.45, 60.23 ± 0.68, 38.20 ± 0.50, and 25.60 ± 0.98 kPa for CPU, CPFSD1, CPFSD2, CPFSD3, and CPFSD4, respectively.

The improved adhesion at moderate SD loading is mainly attributed to the phenolic hydroxyl groups in catechol, which can form hydrogen bonds, coordination bonds, and ligand interactions with tissue functional groups. However, excessive catechol content may cause self-aggregation at the interface or within the matrix, forming local domains that weaken effective interfacial bonding. It may also disrupt the internal interactions of the polyurethane network, affect chain alignment or cross-linking, and thus reduce cohesive strength. Overall, the SD-modified dressings showed a good balance between flexibility and tissue adhesion, providing a solid basis for the subsequent in vivo studies.

### 3.4. Hemostatic/Antioxidant Properties of CPFSD

**Figure 4:**
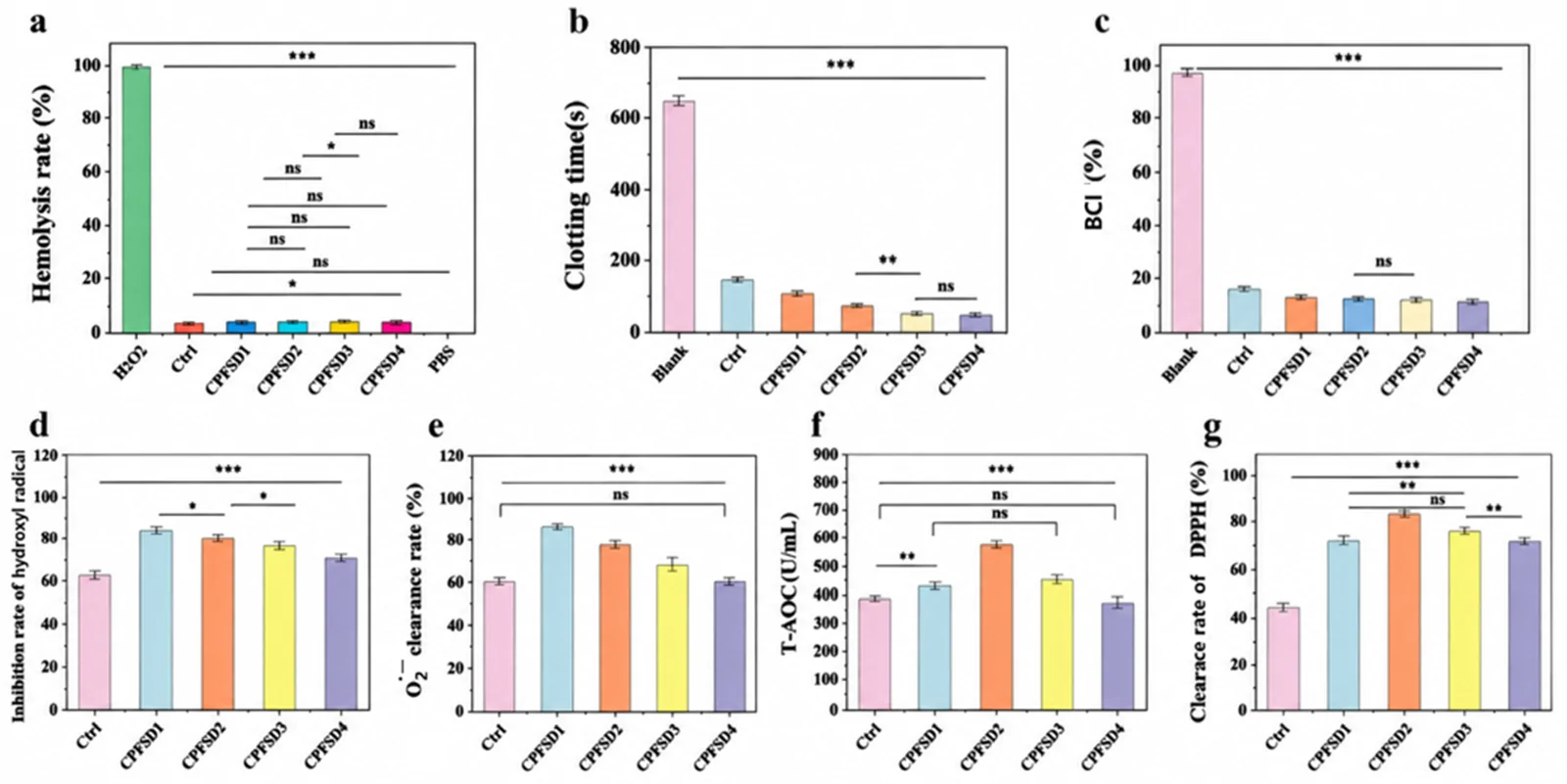
Hemocompatibility, hemostatic performance, and antioxidant activity of the dressings. (**a**,**b**) Hemocompatibility; (**c**) Clotting time; (**d**) Hemostasis process; (**e**) Blood clotting index; (**f**,**g**) Scavenging of O_2_·^−^, DPPH·, ·OH, and T-AOC (Data are expressed as mean ± standard deviation, *n* = 3. ns: not significant, * *p* ≤ 0.05, ** *p* ≤ 0.01, *** *p* < 0.001).

To evaluate the blood compatibility of the developed dressings, red blood cells were co-incubated with the dressing suspensions, using distilled water as the positive control and phosphate-buffered saline (PBS) as the negative control. After incubation, the supernatants of all dressing groups remained clear and stratified, similar to PBS, indicating minimal hemolysis. The hemolysis rates of all dressings remained below 5% (Figure 4a), meeting internationally accepted clinical standards. These results confirm that the developed dressings possess favorable hemocompatibility and are suitable for safe clinical application.

Because hemostasis is the first stage of wound healing, effective blood clotting is essential for hydrogel bioadhesives used in wound care [45]. To investigate the hemostatic performance of the dressings, an in vitro blood clotting time assay was performed (Figure 4b). The clotting times of the blank control, CPU, CPFSD1, CPFSD2, CPFSD3, and CPFSD4 were 672.64 s, 148.47 s, 105.12 s, 73.21 s, 55.15 s, and 47.66 s, respectively. All dressings significantly shortened coagulation time compared with the blank control, demonstrating strong procoagulant activity. This effect was likely due to the abundant hydrophilic groups on the dressing surface, which can rapidly absorb blood components and accelerate clot formation [46]. In addition, the blood coagulation index (BCI) test showed markedly lower BCI values for all dressings than for the controls, further confirming their ability to promote coagulation (Figure 4c).

To assess the reactive oxygen species (ROS)-scavenging capability of the dressings, superoxide anion (O_2_·^−^) and hydroxyl radical (·OH) were selected as representative ROS species. The dressings exhibited concentration-dependent ROS-scavenging activity (Figures S6 and S7). At 250 μg/mL, approximately 50% of O_2_·^−^ was scavenged, indicating SOD-like activity [47]. At the same concentration, CPFSD dressings scavenged more than 60% of ·OH. The overall antioxidant activity was further evaluated using DPPH· and ABTS·^+^ assays. At 500 μg/mL, more than 85% of DPPH· was eliminated, and up to 400 U/mL of ABTS·^+^ was removed. Comparison of the antioxidant capacities showed that CPFSD1 and CPFSD2 exhibited significantly stronger activity than CPU, CPFSD3, and CPFSD4 at the same concentrations (Figure 4d–g). These results identify CPFSD1 and CPFSD2 as the most promising antioxidant dressings. Collectively, the data demonstrate excellent hemocompatibility, strong hemostatic performance, and outstanding ROS-scavenging and antioxidant activity, providing a solid biological basis for the subsequent cellular and animal studies. Although CPFSD1 possessed superior adhesion and comparable antioxidant activity, it exhibited excessive swelling and poor structural stability. In contrast, CPFSD2 achieved balanced properties including moderate swelling, favorable mechanical strength, and antioxidant capacity. Based on these results, CPFSD2, CPU, and PU-Fe were selected for further mechanistic investigation of wound healing.

### 3.5. Antibacterial and Cytocompatibility of CPFSD

**Figure 5:**
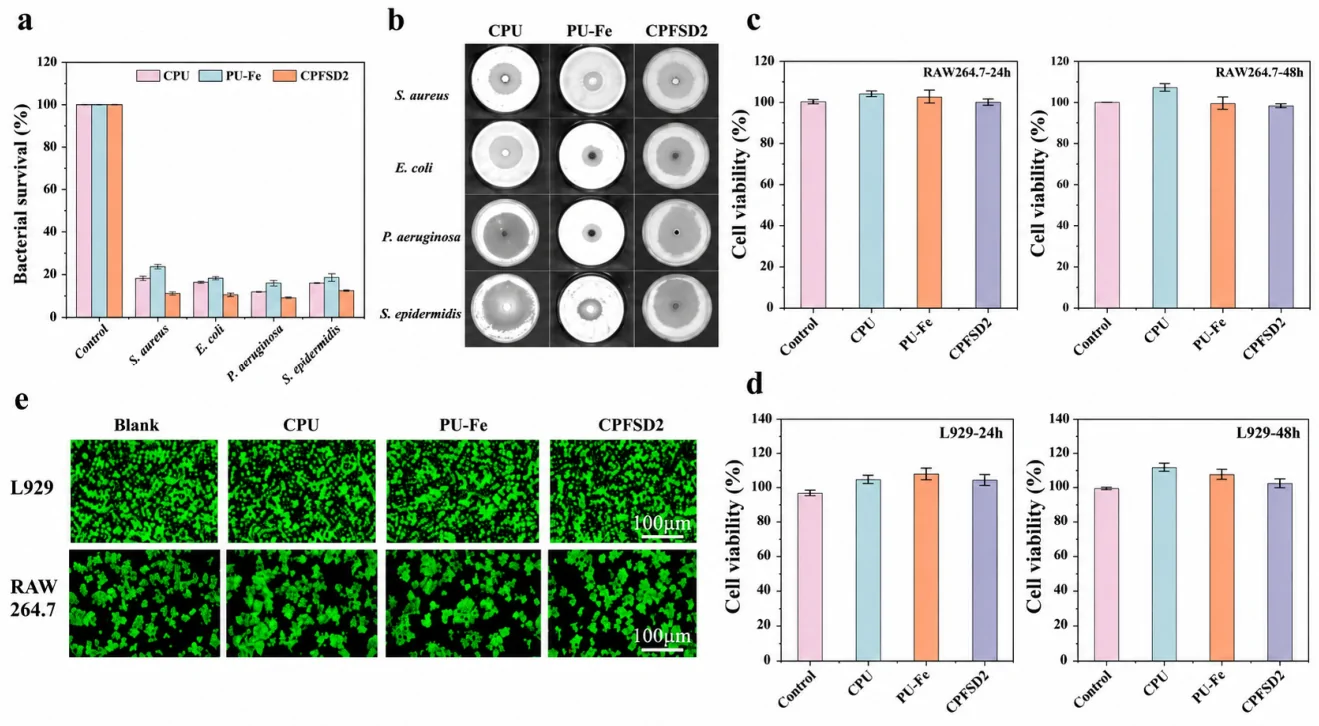
Antibacterial activity and cytocompatibility of the dressings. (**a**,**b**) Antibacterial efficacy against *E. coli*, *S. aureus*, *P. aeruginosa*, and *S. epidermidis*; (**c**,**d**) Cell proliferation of L929 and RAW264.7 cells; (**e**) LIVE/DEAD staining after 48 h incubation (Data are expressed as mean ± standard deviation, *n* = 3).

To evaluate the antibacterial mechanism and the role of curcumin–ferrocene integration, the antibacterial activity of the dressings was first assessed against *S. aureus*, *E. coli*, *S. epidermidis*, and *P. aeruginosa* (Figure 5a). Owing to the interaction between curcumin and iron ions, the dressings exhibited antibacterial activity against all four strains. Within 24 h, the samples containing both curcumin and ferrocene showed strong bactericidal effects, reducing the survival rate of all tested bacteria to below 20%. The combination of curcumin and ferrocene further enhanced the antibacterial performance of the dressings.

Because cytocompatibility is essential for wound dressings applied to damaged tissues, the biological safety of the materials was also investigated. Cytotoxicity and cell proliferation were used as key indicators of biocompatibility. CCK-8 assays showed that L929 fibroblasts and RAW264.7 macrophages exposed to dressing extracts for 24 and 48 h retained more than 99% viability, indicating negligible cytotoxicity (Figure 5c,d). To establish suitable sublethal injury conditions for the cytoprotection assays, the optimal LPS challenge doses were determined to be 200 ng/mL for RAW264.7 cells and 10 μg/mL for L929 cells (Figures S10 and S11). Live/dead staining after 48 h co-culture with dressing extracts showed abundant live cells and only minimal dead-cell staining, indicating good cell survival and proliferation (Figure 5e). Cell density increased over time in the dressing-treated groups, while cell death remained comparable to the control. In addition, diluted dressing extracts did not adversely affect cell viability. Collectively, these results confirm excellent cytocompatibility and support the suitability of the dressings for wound care applications. Overall, the dressings combine broad-spectrum antibacterial activity with strong compatibility toward fibroblasts and macrophages, providing a solid biological basis for the following cellular and animal wound healing studies.

### 3.6. In Vitro Antioxidant/Anti-Inflammatory Effects of CPFSD

Excessive intracellular reactive oxygen species (ROS) accumulation and dysregulated inflammatory responses are key pathological features of non-healing wounds. To determine whether CPFSD can regulate these pathological events, its antioxidant and anti-inflammatory activities were evaluated in L929 fibroblasts and RAW264.7 macrophages under inflammatory conditions. Intracellular ROS levels and pro-inflammatory mediator expression were then quantified.

**Figure 6:**
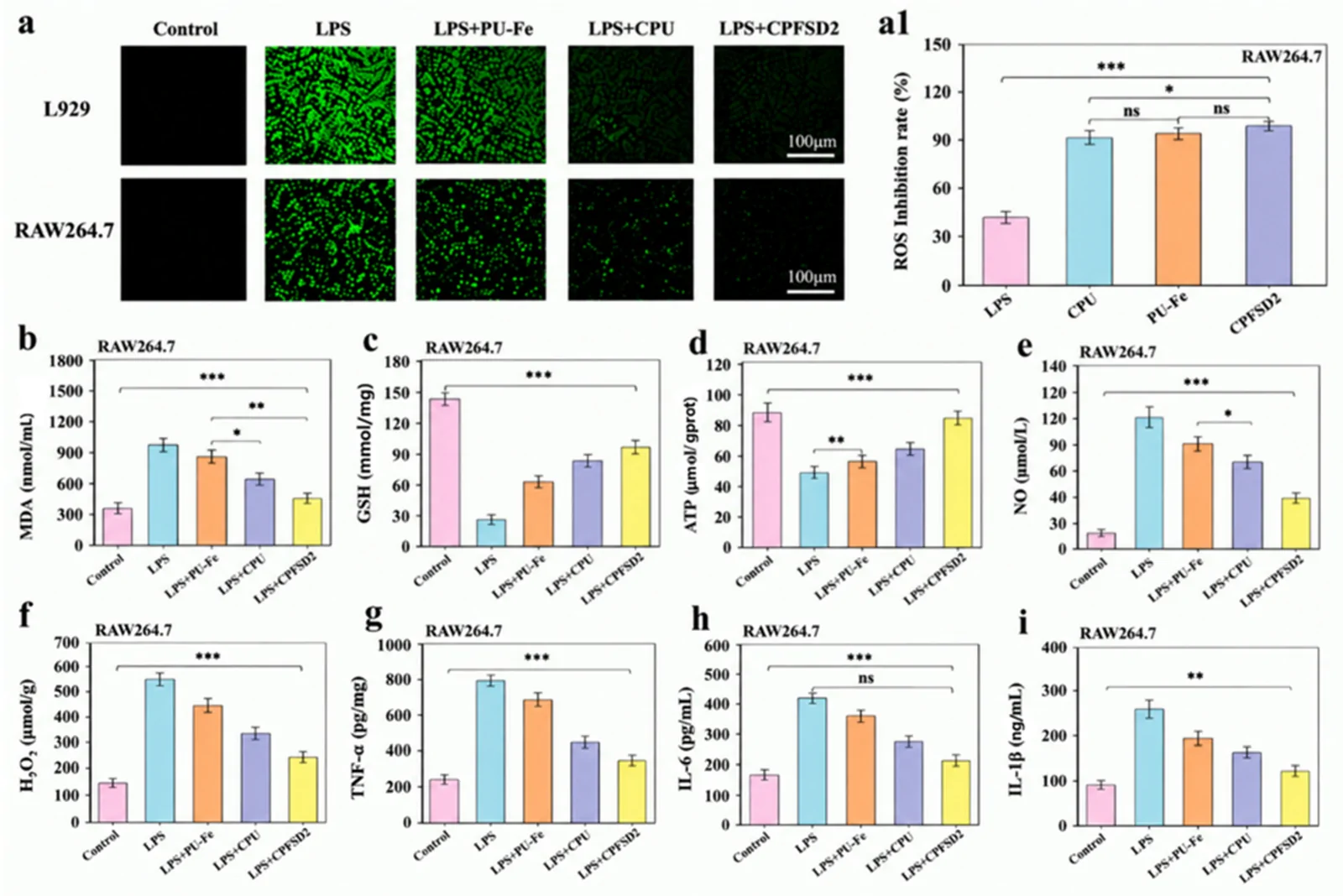
Antioxidant and anti-inflammatory effects in LPS-stimulated RAW264.7 macrophages. (**a**) Intracellular ROS levels; (**a1**) ROS inhibition (DCFH-DA) and synergy analysis; (**b**) MDA levels; (**c**) GSH levels; (**d**) ATP levels; (**e**) NO production; (**f**) H_2_O_2_ levels; (**g**–**i**) TNF-α, IL-6, and IL-1β levels (Data are expressed as mean ± standard deviation, *n* = 3. ns: not significant, * *p* ≤ 0.05, ** *p* ≤ 0.01, *** *p* < 0.001).

To simulate an inflammatory microenvironment, RAW264.7 macrophages and L929 fibroblasts were first stimulated with lipopolysaccharide (LPS), and then treated with CPU, PU-Fe, or CPFSD2. Intracellular ROS levels were measured by DCFH-DA staining. After LPS stimulation, both cell types showed strong green fluorescence, indicating elevated ROS levels. CPU and PU-Fe slightly reduced the fluorescence intensity, whereas CPFSD2 produced the greatest decrease in ROS (Figure 6a). In LPS-stimulated RAW264.7 macrophages, CPU and PU-Fe reduced intracellular ROS by 91.95% and 42.58%, respectively, while their combination achieved 99.98% inhibition. The Bliss independence model predicted an additive inhibition of 95.4%, but the experimental value was higher, confirming a synergistic ROS-scavenging effect under inflammatory conditions. Similar results were observed in LPS-stimulated L929 fibroblasts (Figure 6(a1) and Figure S12a). CPU and PU-Fe suppressed ROS generation by 87.85% and 38.06%, respectively, while CPFSD2 achieved 99.81% inhibition. This experimental value also exceeded the Bliss-predicted additive level of 92.53%, further confirming the synergistic antioxidant interaction. These results show that curcumin and ferrocene work together to regulate ROS in a coordinated and cell-independent manner, supporting the multifunctional therapeutic potential of CPFSD for oxidative stress control during wound healing. This cross-cellular ROS regulation suggests that CPFSD can simultaneously reduce inflammatory oxidative stress and protect regenerative fibroblasts, thereby providing mechanistic support for its improved wound healing performance in vivo.

To further assess oxidative damage, lipid peroxidation was evaluated by measuring malondialdehyde (MDA). LPS stimulation increased MDA to 1055 and 1156 nmol/mL in RAW264.7 and L929 cells, respectively, indicating marked oxidative stress. CPFSD2 reduced MDA to 519 and 522 nmol/mL in the two cell lines, demonstrating strong antioxidative activity (Figure 6b and Figure S12). In addition, CPFSD2 restored intracellular glutathione (GSH) levels after LPS exposure and increased ATP levels toward baseline (Figure 6c,d). Nitric oxide (NO), which regulates macrophage function during wound healing, was also normalized by CPFSD2. The NO level was lower than that in LPS-treated cells but remained higher than that in untreated controls, suggesting a balanced anti-inflammatory effect (Figure 6e). When cells were exposed to exogenous H_2_O_2_, intracellular H_2_O_2_ levels rose sharply in both cell types, but this increase was significantly reduced by CPFSD2, indicating effective protection against oxidative injury (Figure 6f).

Because oxidative stress also influences macrophage polarization, the effect of CPFSD2 on macrophage phenotype was further examined. CPFSD2 promoted a shift toward the M2 phenotype, as shown by significant reductions in TNF-α, IL-1β, and IL-6 levels measured by ELISA (Figure 6g–i). Compared with PU-Fe and CPU at the same dose, CPFSD2 showed superior anti-inflammatory activity. On the basis of its strong antioxidant and anti-inflammatory effects in vitro, CPFSD2 was selected for in vivo evaluation in a full-thickness murine wound model.

### 3.7. In Vivo Wound Healing Effect of CPFSD

**Figure 7:**
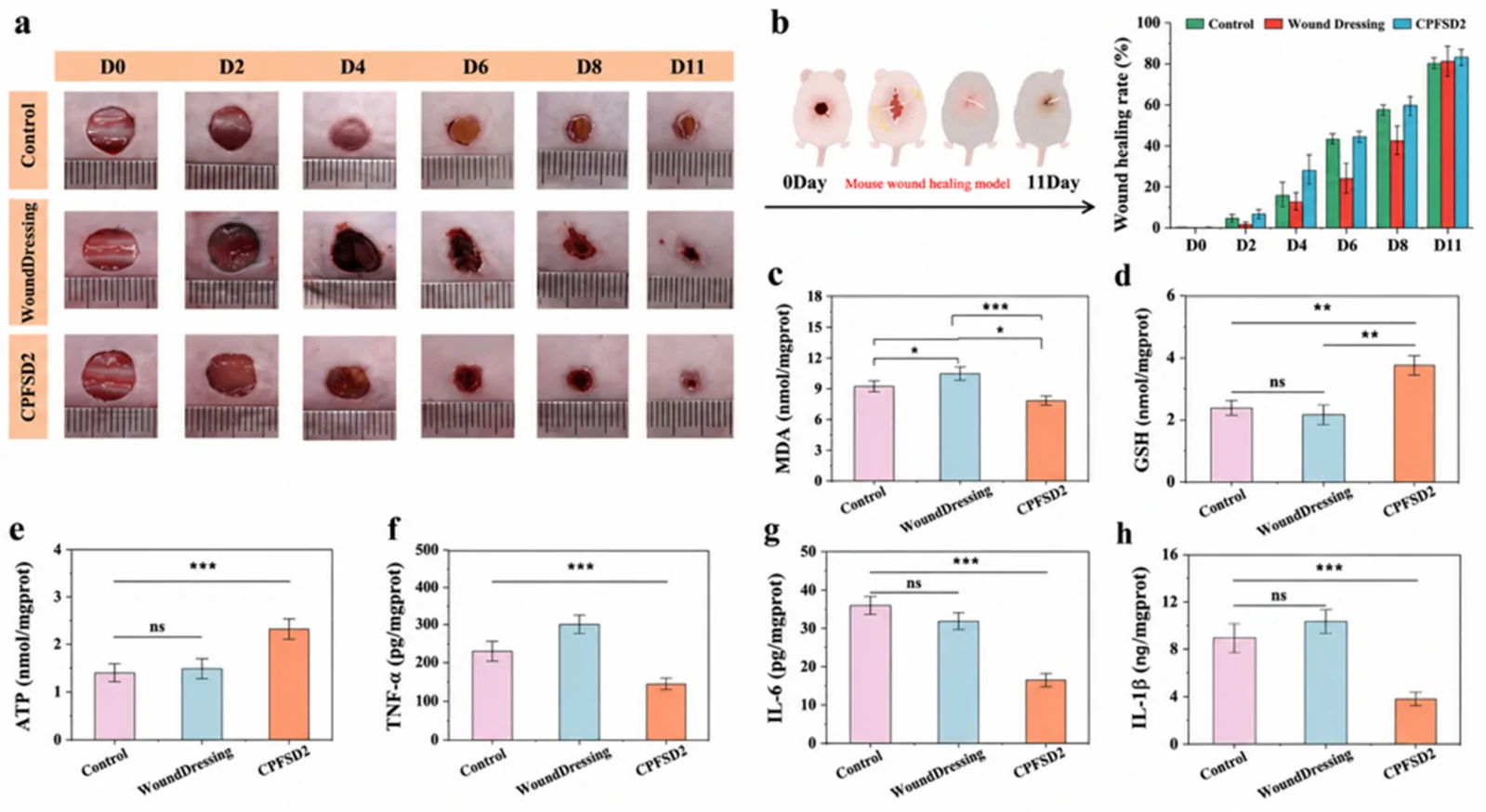
(**a**) Representative wound images at days 0, 2, 4, 6, 8, and 11; (**b**) Quantification of wound closure percentage in mice. (**c**–**e**) Concentrations of malondialdehyde (MDA), glutathione (GSH), and adenosine triphosphate (ATP) in wound tissue homogenates; (**f**–**h**) Levels of pro-inflammatory cytokines TNF-α, IL-6, and IL-1β in wound tissues following different treatments (Data are expressed as mean ± standard deviation, *n* = 5. ns: not significant, * *p* ≤ 0.05, ** *p* ≤ 0.01, *** *p* < 0.001).

Based on the above results, which show that CPFSD2 creates a favorable wound microenvironment through the synergistic antioxidant and anti-inflammatory actions of curcumin and ferrocene, in vivo studies were further performed to evaluate its effects on wound closure, epidermal regeneration, extracellular matrix deposition, and angiogenesis. Wound healing was monitored for 11 days. Representative images on days 2, 4, 6, 8, and 11 are shown in Figure 7a. By day 11, the CPFSD2 group exhibited a tendency toward a higher wound-closure rate compared with the other groups. The wound closure percentages on day 11 were (81.4 ± 2.08)% for the control, (81.9 ± 6.94)% for wound dressing, and (84.1 ± 3.26)% for CPFSD2, although no statistically significant difference was detected among the three groups. These results suggest a trend of accelerated wound closure upon CPFSD2 treatment.

To further clarify the in vivo mechanism, representative biomarkers related to oxidative damage (MDA), glutathione (GSH), energy metabolism (ATP), and inflammatory response (TNF-α, IL-6, and IL-1β) were systematically analyzed in wound tissues. This was performed to comprehensively evaluate the therapeutic performance of CPFSD in vivo. GSH levels were significantly higher in CPFSD2-treated mice than in the control group (Figure 7d). ATP levels were also significantly increased after CPFSD2 treatment (Figure 7e). MDA, a marker of lipid peroxidation and oxidative damage severity, was markedly reduced by CPFSD2. The MDA concentrations in wound tissues were 9.23 ± 0.56, 10.87 ± 1.01, and 7.57 ± 0.40 μmol/mg protein for the control, wound dressing, and CPFSD2 groups, respectively (Figure 7c). These results indicate that CPFSD2 promoted wound healing while alleviating oxidative stress in vivo.

To evaluate the inflammatory response during healing, TNF-α, IL-1β, and IL-6 levels in wound tissues were quantified by ELISA (Figure 7f–h). Consistent with the gross inflammatory appearance, the control and wound dressing groups showed elevated cytokine levels. In contrast, CPFSD2 treatment significantly reduced TNF-α, IL-1β, and IL-6 concentrations. Collectively, these findings demonstrate that CPFSD2 accelerated wound healing in mice by reducing oxidative stress, suppressing inflammatory cytokine production, and decreasing cellular apoptosis.

**Figure 8:**
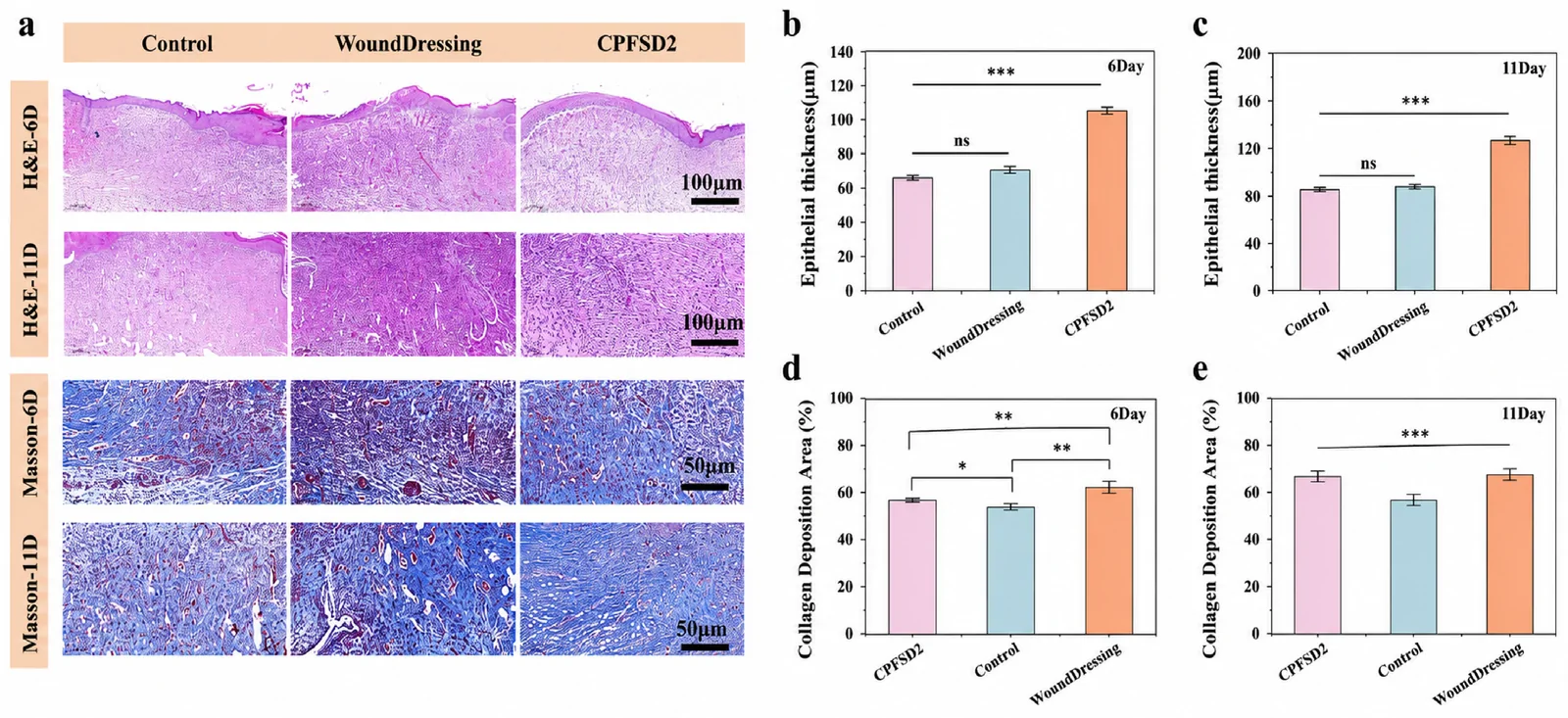
(**a**) Representative images of H&E staining (scale bar: 100 µm) and Masson’s trichrome staining (scale bar: 50 µm); (**b**,**c**) Quantification of epidermal thickness at days 6 and 11; (**d**,**e**) Quantification of collagen deposition area at days 6 and 11 (Data are expressed as mean ± standard deviation, *n* = 5. ns: not significant, * *p* ≤ 0.05, ** *p* ≤ 0.01, *** *p* < 0.001).

To further evaluate the quality of tissue regeneration at the wound site, hematoxylin and eosin (H&E) staining and Masson’s trichrome staining were performed to examine inflammatory cell infiltration, re-epithelialization, and collagen deposition during wound repair. On day 11 after injury, re-epithelialization was observed in all groups. However, the CPFSD2-treated wounds exhibited a more continuous epidermis that was well aligned parallel to the skin surface. Epidermal thickness at days 6 and 11 is summarized in Figure 8b,c. On day 6, the mean epidermal thicknesses were 87.1 ± 1.6, 89.0 ± 1.9, and 134.9 ± 3.8 µm in the control, wound dressing, and CPFSD2 groups, respectively. On day 11, the corresponding values were 67.4 ± 1.1, 71.5 ± 2.2, and 106.4 ± 1.5 µm, respectively. These results indicate that CPFSD2 markedly accelerated epidermal regeneration compared with the control.

Histological observation on day 11 also showed increased fibroblast proliferation, collagen deposition, and neovascularization in the dressing-treated groups compared with the control. In contrast, the control wounds displayed obvious inflammatory cell infiltration. CPFSD2 treatment reduced inflammation, promoted fibroblast activity, enhanced collagen deposition, and supported more orderly tissue repair. Masson’s trichrome staining further confirmed that collagen deposition was significantly greater in CPFSD2-treated wounds on days 6 and 11 than in the control group. Collectively, these findings demonstrate that CPFSD2 improved the histological quality of wound healing.

**Figure 9:**
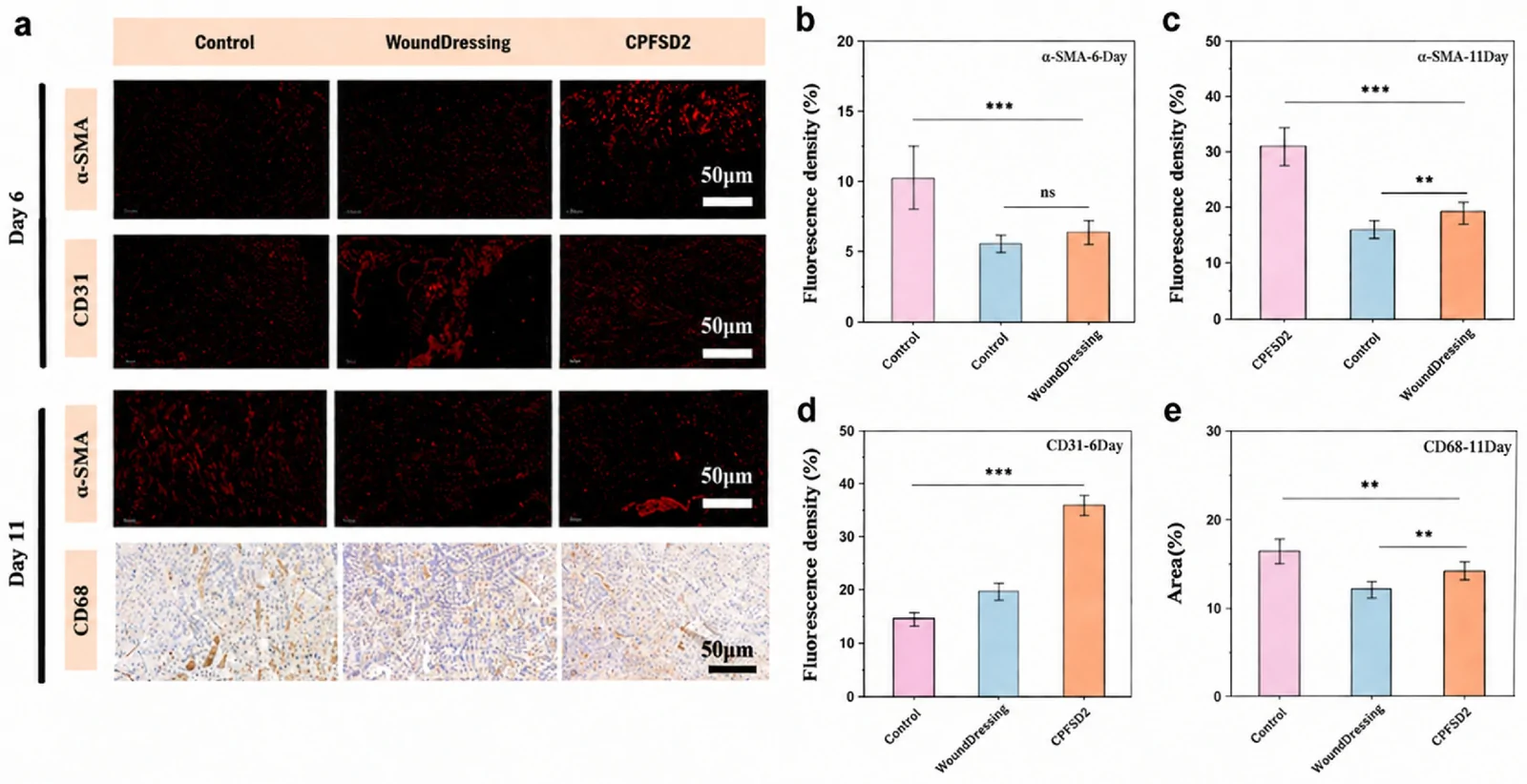
(**a**) Representative immunofluorescence images of α-SMA, CD31, and CD68 in wound sections at days 6 and 11 (scale bar: 50 µm); (**b**–**e**) Quantification of fluorescence intensities forα-SMA, CD31, and CD68 in the wound bed (Data are expressed as mean ± standard deviation, *n* = 5. ns: not significant,** *p* ≤ 0.01, *** *p* < 0.001).

To further explore the immunomodulatory and pro-angiogenic effects of CPFSD2, in vivo immunofluorescence staining was performed after wound treatment. On day 6 post-injury, staining for CD31, an endothelial marker, and α-SMA, a mature-vessel marker, showed much stronger signals in CPFSD2-treated wounds than in the control groups (Figure 9a,b,d. This indicates higher expression of vascular markers and effective promotion of neovascularization. By day 11, the α-SMA-positive area in the CPFSD2 group was significantly larger than that in the other groups, suggesting continued maturation of newly formed vessels (Figure 9c). α-Smooth muscle actin is also expressed by myofibroblasts and supports cell contractility and cytoskeletal organization during tissue remodeling. Its elevated expression helps reduce intercellular gaps and accelerate wound closure, which is consistent with the faster healing observed in the CPFSD2-treated wounds.

CD68 was then examined to assess inflammatory status. As a transmembrane glycoprotein enriched in monocyte-macrophages, CD68 is widely used as a marker of macrophage activation and inflammation [48]. Its localization on plasma, endosomal, and lysosomal membranes also reflects phagocytic activity. Quantitative immunostaining showed CD68-positive cell percentages of (16.95 ± 1.14)% in the control group, (12.12 ± 0.30)% in the wound dressing group, and (14.67 ± 0.50)% in the CPFSD2 group (Figure 9e). Both the wound dressing and CPFSD2 groups exhibited lower proportions of CD68-positive macrophages than the untreated control, suggesting reduced macrophage-associated inflammatory infiltration at day 11. Notably, the wound dressing group showed the lowest CD68-positive cell proportion among the three groups. This difference may reflect distinct inflammatory-resolution kinetics between the two treatments. Overall, these results indicate that both dressings modulated macrophage-associated inflammation relative to the untreated control, while the difference between the wound dressing and CPFSD2 groups may reflect distinct temporal patterns of macrophage response during wound healing. In addition, H&E staining of major organs, including the heart, liver, spleen, lung, and kidney, confirmed the systemic biocompatibility of CPFSD2 (Figure S13).

Overall, CPFSD2 exhibited a trend toward faster wound closure compared with the other groups. Its favorable regenerative performance can be mainly attributed to two factors. First, its strong tissue adhesion ensured close contact with the wound bed, which helped seal the defect and maintain a protective barrier. Second, curcumin and ferrocene reduced inflammatory cell infiltration, enhanced collagen deposition, promoted granulation tissue formation, and accelerated re-epithelialization. Together, these effects make CPFSD2 a highly promising wound dressing for tissue repair.

## 4. Conclusions

In summary, we developed a curcumin-functionalized polyurethane composite dressing (CPFSD) by co-integrating curcumin, ferrocene (Fc), and dopamine-grafted sodium alginate (SD) within a polyurethane matrix, thereby realizing a rational “synergistic therapy coupled with secure adhesion” design paradigm. Mechanistically, curcumin provides intrinsic antioxidant and anti-inflammatory activity while Fc augments ROS scavenging via reversible Fe^2+^/Fe^3+^ redox cycling; concurrently, catechol/hydroquinone motifs on SD mediate stable interfacial bonding and modulate Fc retention. These complementary functionalities act in concert to attenuate oxidative stress, suppress pro-inflammatory signaling, and maintain intimate dressing-tissue contact under mechanically demanding conditions. Comprehensive physicochemical characterization, in vitro cell-based assays, and an in vivo wound model collectively demonstrate that CPFSD promotes tissue regeneration and exhibits favorable biocompatibility. Overall, CPFSD represents a promising candidate and a compelling design framework for the development of clinically translatable advanced wound dressings tailored to complex pathological wounds.

## Data Availability

The original contributions presented in this study are included in the article/Supplementary Material. Further inquiries can be directed to the corresponding author.

## Supplementary Materials

The following supporting information can be downloaded at: https://www.mdpi.com/article/10.3390/jfb17090473/s1. Section S1.1. Characterization of CPFSD dressings; Section S1.2. Physical properties of CPFSD dressings; Section S1.3. Evaluation of mechanical properties; Section S1.4. Evaluation of adhesive properties; Section S1.5. In vitro coagulation properties; Section S1.6. In vitro biological evaluation; Section S1.7. In vivo experiment; Table S1. Compositional details of CPU, PU-Fe, and CPFSD-series composite dressings; Figure S1. Promoting effects of water, CPFSD, and FeSO_4_ on hydroxyl radicals in a short time; Figure S2. XPS of CPFSD; Figure S3. SEM images of CPU, CPFSD1, CPFSD2, CPFSD3, and CPFSD4; Figure S4. UV-Vis of CPFSD; Figure S5. Cumulative curcumin release profiles. (pH = 6.5); Figure S6. Effects of CPU, CPFSD1, CPFSD2, CPFSD3, and CPFSD4 dressings on the elimination of O_2_·^−^ at different concentrations; Figure S7. Effects of CPU, CPFSD1, CPFSD2, CPFSD3, and CPFSD4 dressings on the elimination of hydroxyl radicals (·OH) at different concentrations; Figure S8. Effects of CPU, CPFSD1, CPFSD2, CPFSD3, and CPFSD4 dressings on the elimination of DPPH· at different concentrations; Figure S9. Effects of CPU, CPFSD1, CPFSD2, CPFSD3, and CPFSD4 dressings on the elimination of total antioxidant capacity (T-AOC) at different concentrations; Figure S10. Different concentrations of LPS-induced RAW264.7; Figure S11. Different concentrations of LPS-induced L929; Figure S12. ROS-scavenging capacity of L929 cells under CPFSD dressing treatment; Figure S13. HE staining of heart, liver, spleen, lung, and kidney in mice with CPFSD2 dressing.
