# Supplementary material for: Curcumin (Ferrocene)-Functionalized Polyurethane Composite Dressing Integrated with Dopamine-Grafted Sodium Alginate: Synergistic ROS Modulation and Durable Tissue Adhesion
Source: J Funct Biomater. 2026 Sep 17;17(9):473. doi: 10.3390/jfb17090473 (PMC13608592; doi:10.3390/jfb17090473)
Supplement: Supplementary file 1 [file jfb-17-00473-s001.zip › jfb-4514692-supplementary.pdf]

Curcumin (Ferrocene)-Functionalized Polyurethane Composite Dressing Integrated with Dopamine-Grafted Sodium Alginate: Synergistic ROS Modulation and Durable Tissue Adhesion

S1. Supporting methods

S1.1. Characterization of CPFSD dressings

A ZEISS Sigma 360 field emission scanning electron microscope (FESEM) was used to observe the internal structure of the CPFSD. Fourier transform infrared (FT-IR) spectra of SA-DA, CPU, and CPFSD were recorded in the wavenumber range of 4000 cm^{-1} to 500 cm^{-1}(IRPrestige-21, Shimadzu, Japan). The chemical structures of CPFSD were analyzed using X-ray photoelectron spectroscopy (XPS, Thermo Scientific ESCALAB 250Xi). The structures of CPFSD were characterized by X-ray diffraction. The characteristic absorption peaks of SA-DA were measured using a UV-Vis spectrometer(UV-2600, Shimadzu, Japan). SD was dissolved in D_2O for ^1H-NMR characterization.

S1.2. Physical properties of CPFSD dressings

S1.2.1. Swelling rate

The CPFSD was shaped into a square with a length of 10 mm, then freeze-dried and further dried in an oven at 40°C until it reached a constant weight. The side length C1 after drying was measured. Subsequently, the dried CPFSD was immersed in Petri dishes filled with deionized water at 37 °C. Following a set time interval, the CPFSD was retrieved from the water, and any excess surface moisture was carefully absorbed with filter paper. The side length C2 of the CPFSD was measured. The water swelling rate of the CPFSD was calculated by the equation:

Swelling rate = (C2/C1) ×100%.

This experiment was repeated three times.

S1.2.2. Water absorption

To produce the dry CPFSD, we first molded them into squares with a length of 10 mm. Then, we freeze-dried the CPFSD until they achieved a consistent weight. The mass of the CPFSD post-drying was recorded as W0. Subsequently, the dried samples were

placed in a Petri dish filled with deionized water. Periodically, each CPFSD was removed from the water. Gently wipe off any excess surface water with filter paper. The weight of the CPFSD was measured and recorded as mass W1 after water absorption. The water absorption was calculated using the equation:

Water absorption rate = [(W1 - W0) / W0] × 100%

We conducted three parallel measurements for each CPFSD.

S1.2.3. Contact angle measurements

We measured the contact angle of all the samples at ambient temperature for at least five independent measurements (SL200L2). Samples with the size of 10 × 10 mm were placed on the testing plate before different liquids were dropped onto the surface. The volume of liquid droplets was controlled at 1 μL.

S1.2.4. Measurement of water vapor transmission rate

Fill a glass bottle with a 13 mm diameter with water. Cover it with CPFSD and seal it with sealing CPFSD. Record the initial mass, Wi. The glass vial, equipped with the composite dressing, was then placed in a controlled environment chamber maintained at 37 °C and 35% relative humidity for 24 hours. After 24 hours, the mass of the vial was measured again and recorded as Wt. A small glass vial containing only ultrapure water was used as a blank control. The following formula was used to determine the water vapor transmission rate:

$$\mathrm{WVTR} = \frac{\mathrm{Wi} - \mathrm{Wt}}{\mathrm{A} \times \mathrm{t}}$$

where WVTR is expressed in g/(m^2·day), Wi and Wt are the masses of water in the glass vials before and after being placed in a constant temperature and humidity incubator at 37°C and 35% humidity, A is the area of the glass vial mouth (m^2), and t is the duration of the measurement.

S1.2.5. Thermal stability analysis

The CPFSD was analyzed for thermal stability using TG (TA).

S1.2.6. Evaluation of drug release properties

The in vitro drug release behavior of the CPFSD was examined.. The CPFSD was placed in a centrifuge tube containing 20 mL of PBS buffer solution with a pH of 6.5. The tube was then shaken in a constant-temperature oscillator at 37 °C. At specified intervals, 3 milliliters of solution was removed from the test tube and replaced with 3 mL of buffer solution. This process was repeated at each interval. The solution removed

from the centrifuge tube was analyzed for absorbance at 425 nm using a UV-Vis spectrophotometer, and the Cur content in the release solution was determined based on a standard curve.

S1.2.7. Evaluation of degradable properties

The in vitro degradation properties of the CPFSD were tested·. Prepare squares with 10mm side length. Then, freeze-dried and weighed at a mass of W0. Afterward, the lyophilized CPFSD were immersed in PBS solution (pH 7.4) and lysozyme solution with a concentration of 2 μg/mL on an air shaker (temperature: 37 °C, speed: 120 rpm). Subsequently, the samples were exchanged with an equal amount of fresh PBS solution and lysozyme solution of the same concentration every 3 days. The samples were retrieved, cleansed, lyophilized, and weighed on the 1st, 3rd, 5th, and 14th days. Samples were then retrieved, cleansed, lyophilized, and weighed. The degradation (W) of CPFSD was calculated according to the following equation:

$W=(1-Wx/W0) \times 100\%$

where W0 is the initial mass of CPFSD after freeze-drying, and Wx is the remaining mass of CPFSD at different times. Each set of experiments was repeated three times.

S1.2.8. Ferrocene-Induced ROS generation experiment

The hydroxyl radical (·OH) generation capacity of ferrocene-modified polyurethane dressings was determined by the salicylic acid method combined with the Fenton reaction principle. Specifically, the Fe^{2+} released from the dressings reacts with H_2O_2 to generate ·OH, which can attack salicylic acid molecules and produce 2,3-dihydroxybenzoic acid with characteristic absorption at 510 nm. The content of ·OH was then quantified based on the absorbance value. For the experiment, 6 mmol/L salicylic acid solution, 10 mmol/L H_2O_2 solution, and 10 mg/mL dressing extract were prepared. Experimental groups, control groups, blank groups, and negative control groups were set up (with 3 replicates per group). After preheating at 37 °C and reacting for 1 h in the dark, the absorbance was measured using deionized water as the reference. The ·OH generation effect was evaluated according to the criterion that A_{510} of the experimental group > A_{510} of the control group > A_{510} of the blank group, while A_{510} of the negative control group was close to 0. Throughout the experiment, it was necessary to strictly control the timeliness of reagents, reaction temperature, and light-shielding conditions, and avoid contamination interference to ensure the reliability of the results.

S1.3. Evaluation of mechanical properties

S1.3.1. Tensile test

The rectangular CPFSD measures 20 mm by 40 mm by 1 mm. The tensile performance of these CPFSDs was tested using a universal testing machine (Dongguan GOTECH Testing Equipment Company). The tensile test was conducted at a speed of 50 mm/min.

S1.4. Evaluation of adhesive properties

S1.4.1. Lap shear test

The lap shear tests were conducted using a universal testing machine (Dongguan GOTECH Testing Equipment Company) to evaluate the adhesion strength of CPFSD on various material surfaces. The prepared CPFSD samples were placed between two pieces of fresh pig skin, each covering an area of 20 mm × 20 mm. The peel strength of the samples was measured at a constant peeling rate of 50 mm/min. Additionally, the adhesion of the CPFSD to glass, wood, rubber, and metal surfaces was evaluated, with each test repeated at least three times to ensure accuracy. The adhesion strength was calculated using the equation:

Adhesion Strength =F/S

In the equation, F represents the maximum tensile stress (N), and S represents the area of CPFSD adhesion (m^2).

S1.4.2. Peel test

For the peel test of CPFSD adhered to soft tissue, pig skin with an adhesive area of 20 mm width and 20 mm length was tested by the standard tensile test (ASTM F2258). In brief, CPFSD was applied to the overlap area of the sample tissue. The peel tests were then conducted at a controlled rate of 50 mm/min, and the load-force curve was recorded until adhesive failure. Tensile strength was quantified by dividing the maximum adhesion force by the bond area, and each sample was tested three times to ensure the accuracy and reliability of the results.

S1.5. In vitro coagulation properties

S1.5.1. Whole blood clotting index

To evaluate the film's ability to promote blood clotting, the blood clotting index (BCI), representing the whole blood-clotting rate, was measured using the following procedure.. In brief, 100 μL of citrated whole blood (CWB) from a rat was dropped onto a CPFSD surface and incubated for 7 min at 37 °C. Then, the membrane was placed in 10 mL of deionized water (DIW), and the optical density (OD) of the solution at 540 nm was determined using a versatile microplate reader. A solution composed of DIW and CWB unexposed to CPFSD was used as a reference, and its OD value was represented as 100%. BCI values were calculated using the following formula:

BCI = $Abs_{sample}/Abs_{blank} \times 100\%$

where Abs_{sample} represents the absorbance of the sample group, and Abs_{blank} represents the absorbance of the blank group.

S1.5.2. In vitro clotting time

To start the process, put 10 mg of the sample into a 2-ml plastic tube. Then, it was incubated at 37°C for 10 minutes. Fresh blood containing sodium citrate was incubated at 37°C for 10 min. Following this, 200 μL of the incubated blood was introduced into the sample tube, and 10 μg of the 0.17 M $CaCl_2$ solution was added. The clotting process was carefully monitored in the incubator at 37°C. Gently rotate the sample tube every 10 seconds until the blood stops flowing visibly, which indicates that a clot has formed. The time taken for clotting to occur was accurately recorded. Be sure to repeat the experiment at least three times for each sample.

S1.6. In vitro biological evaluation

S1.6.1. Antioxidant capacity

Different groups of CPFSD samples, each weighing 0.1 g, were cut into pieces and placed in test tubes. To each test tube, 5 mL of 80% methanol was added. The samples were incubated in an ice-water bath for 30 minutes and subsequently centrifuged at 12,000 rpm for 10 minutes to collect the supernatant for further analysis. The dressings were then treated using the appropriate kit, and absorbance measurements were taken at 517 nm, 550 nm, and 520 nm using a spectrophotometer.
The DPPH clearance was calculated using the following formula:

Clearance of DPPH (%)= $1-(\frac{A1-A0}{A2}\times 100\%)$

where A0 is the absorbance of the sample solution mixed with solvent; A1 is the absorbance of the sample after reaction with DPPH; and A2 is the absorbance of the DPPH solution mixed with solvent.
The hydroxyl radical scavenging rate formula was as follows:

Hydroxyl radical scavenging rate (%)= $\frac{Acontrol-Asample}{Acontrol-Ablank}\times 100\%$

where Asample represents the absorbance of the assay tube; Acontrol represents the absorbance of the control tube; and Ablank represents the absorbance of the blank tube.
The total antioxidant capacity formula was as follows:

T-AOC $=\frac{Aa-Ab}{0.01}\div T\times\frac{v1}{v2}\times N$

where Aa represents the absorbance of the assay tube; Ab represents the absorbance of the control tube; T represents the reaction time, 30 min; v1 represents the total volume of the reaction system, mL; v2 represents the volume of the sample, mL; and N represents the number of times the sample was diluted before testing.
The formula for superoxide anion removal rate was as follows:

Superoxide anion removal rate= $\frac{Acontrol-Asample}{Acontrol}\times 100\%$

where Asample represents the absorbance of the assay tube; Acontrol represents the absorbance of the control tube.

S1.6.2. Bacterial viability rate test

Expose the dressing to ultraviolet (UV) light for 2 hours. Afterward, the dressings were transferred into sterilized LB liquid medium. Add 100 μL of a bacterial suspension containing Staphylococcus aureus, Pseudomonas aeruginosa, Staphylococcus epidermidis, and Escherichia coli (1×10^6 CFU/mL) to each test tube. The tubes were secured on a shaker and incubated at 37° C and 100 rpm for 24 hours. At the end of the incubation period, a sample of the bacterial suspension was collected, and the absorbance at 600 nm was measured using a UV-Vis spectrophotometer. The formula for calculating the survival rate of bacteria was as follows:

Bacterial viability (%)= $\frac{ODsample-ODblank}{ODcontrol-ODblank}\times 100\%$

where ODsample represents the OD600 values of composite dressings; ODcontrol represents the OD600 values of LB medium containing bacterial suspensions; and ODblank represents the OD600 values of liquid LB.

S1.6.3. Antimicrobial circle test

The antibacterial activity of the CPFSD was assessed using Staphylococcus aureus (S. aureus), Escherichia coli (E. coli), Pseudomonas aeruginosa (P. aeruginosa), and

Staphylococcus epidermidis (S. epidermidis). Prior to the experiment, the samples were placed in a UV sterilizer for 2 hours in a clean bench and then set aside. The four bacterial strains were evenly spread on LB solid medium, and a 5 mm round CPFSD of the dressing was placed in the center of the plate. The diameter of the inhibition zone was measured after incubation at 37°C for 18 hours.

S1.6.4. Assessment of cytocompatibility

The CCK-8 method was used to qualitatively evaluate the cell proliferation rate of the CPFSD extract liquid. Experimental cells were mouse fibroblasts (L929) and mouse monocyte macrophage leukemia cells (Raw264.7). Initially, the CPFSD was sterilized with UV irradiation. Next, a cell suspension (100μL/well) was seeded in a 96-well plate, and the plate was pre-cultured in an incubator for 24 h. Subsequently, the concentrations of 10, 30, 60, and 120 μg /mL of CPFSD extract liquids were added to the plate and incubated for 24 hours. Following this, the CCK-8 cytotoxicity assay was performed, and the absorbance at 450 nm was measured using an enzyme-labeled instrument. Each group underwent five parallel trials.

The LIVE/DEAD staining method was applied to investigate the cytotoxicity of the CPFSD. 1.0 mL of mouse fibroblasts (L929, density = 5,000 cells mL^{-1}) and Mouse monocyte macrophage leukemia cells (Raw264.7, density = 5,000 cells mL^{-1}) were exposed to concentrations of 10, 30, 60, and 120 μg /mL of CPFSD extract liquid and cultured for 48 h. At predetermined time points, the cells were stained with calcein acetoxymethylester/propidium iodide (calcein-AM/PI) double stain kit for 10 min. Fluorescent images were acquired using an FV1200 confocal microscope (Olympus Corporation, Japan).

S1.6.5. Measurements of cytokines

After seeding RAW264.7 cells and L929 cells at a density of 1×10^6 cells per well in six-well plates and allowing overnight adherence, CPFSD2 (30 μg mL^{-1}), CPU (30 μg mL^{-1}), and PU-Fe (30 μg mL^{-1}) were individually added to the wells for a 1 h pretreatment. Subsequently, the cells were stimulated with LPS (final concentration: 10 μg mL^{-1} for L929 cells; 200 ng mL^{-1} for RAW264.7) for 24 h. Following supernatant collection from each group, the concentrations of NO, MDA, GSH, ATP, TNF-α, IL-6, and IL-1β were determined using an ELISA assay.

S1.6.6. Intracellular ROS detection

Intracellular ROS generation was assessed using a DCFH-DA assay. RAW264.7 cells and L929 cells were seeded at a density of 1×10^6 cells per well in six-well plates and allowed to adhere overnight. CPFSD2 (30 μg mL^{-1}), CPU (30 μg mL^{-1}), and PU-Fe (30 μg mL^{-1}) were added to the wells for a 1 h pretreatment. Subsequently, the cells were

stimulated with LPS (final concentration: 10 μg mL^{-1} for L929 cells; 200 ng mL^{-1} for RAW264.7) for 24 h. The culture medium was then discarded, and the DCFH-DA probe was treated with 50 μg/ml Rosup for 30 minutes. This was followed by one wash with PBS and then detection by laser confocal imaging.

Intracellular ROS levels (DCFH-DA staining) were quantified from confocal images using ImageJ (Fiji). Mean fluorescence intensity per cell was measured with identical acquisition and analysis settings across groups. ROS inhibition was calculated as E=(Fmodel−Fsample)/Fmodel

where Fmodel and Fsample denote mean fluorescence intensities of the LPS model and treatment groups, respectively. Synergistic interactions between curcumin and ferrocene were evaluated using the Bliss independence model:

Eexpected=ECPU+EPU-Fe−ECPU×EPU-Fe

Observed inhibition exceeding Eexpected was considered indicative of synergy.

S1.6.7. Assessment of hemolysis

The anticoagulated whole blood obtained was first centrifuged at 3000 rpm for 10 min to separate the red blood cells. Post-centrifugation, the red blood cells were repeatedly washed with PBS solution until the supernatant became clear, and the supernatant was discarded to collect the purified red blood cells. An appropriate amount of these purified red blood cells was then mixed into a freshly prepared PBS solution to create a red blood cell suspension with a 2% (v/v) volume concentration. Simultaneously, 5 mg of CPFSD was immersed in 2 mL of PBS solution at 37°C for 24 h to obtain the sample eluate.125 μL of the sample eluate was thoroughly mixed with 875 μL of the red blood cell suspension. This mixture was then incubated at 37°C for 1 h and subsequently centrifuged at 3000 rpm for 10 min. Then, 100 μL of the supernatant was carefully transferred to a 96-well plate. The absorbance at 540 nm was measured using a multifunctional enzyme-linked immunosorbent assay (ELISA) reader. The positive and negative controls used were H_2O and PBS solution, respectively. The rate of hemolysis was calculated by the equation:

Rate of Hemolysis = $(Abs_{sample}-Abs_{negative})/(Abs_{Positive}-Abs_{negative})\times100\%$

Among them, Abs_{sample} , $Abs_{negative}$, and $Abs_{Positive}$ correspond to the absorbance values of the sample, negative control group, and positive control group, respectively

S1.7. In vivo experiment

S1.7.1. Wound healing in a model for full-thickness skin defect

The animals used in the experiment were provided with standard water and food. Specific conditions of 20–22 °C and 50–60% humidity were maintained for these experimental animals. From a physiological perspective, male animals exhibit more

stable hormone levels compared to females. By using male animals in this study, we aim to focus on the mechanisms of wound healing and the effects of interventions, minimizing the potential confounding factors introduced by physiological differences between sexes. This approach ensures more targeted and clear results. During the experimental design phase, we prioritized investigating the impact of novel dressings on the wound healing process, tissue repair quality, and related biomarkers, all of which can be clearly and stably observed in male animal models. Therefore, we believe that the sex of the animals has limited and controllable influence on the study's results and will not affect the accurate assessment of wound healing mechanisms and the efficacy of interventions. The full-thickness skin defect model is a widely recognized animal model for assessing wound healing capability. Mice were allocated into a control group(cyanoacrylate group), a commercially available wound dressing group, and a CPFSD group. Each mouse had a wound of 10 mm diameter created on its back, after which the respective CPFSD was applied to the wound area. The healing process of the wound was closely monitored and documented with photographs on postoperative days 2, 4, 6, 8, 10, and 14. The wound area was quantitatively analyzed using ImageJ 5.0 software, and all measurements were normalized to the initial wound area (100%) to ensure accurate comparisons. To gain a deeper insight into the healing progression, tissue samples were harvested from the areas of wound healing. These samples were then subjected to staining with hematoxylin and eosin (H&E) and Masson's trichrome stain, facilitating a detailed histological analysis of the healing process. The hearts, livers, spleens, lungs, and kidneys of the mice were stained and used to assess the non-toxicity of the dressings.

S1.7.2. In vivo therapeutic outcome of dressing in mice

All mice were procured from Hangzhou Qizhen Laboratory Animal Science and Technology Co. in China. In order to evaluate the effect of dressing treatment on wound healing, a randomized controlled trial was conducted. The mice were divided into six groups of five mice each. Subsequently, the mice were euthanized, and skin tissues were collected for the detection of biomarkers using various ELISA kits. Levels of inflammatory factors, including MDA, GSH, ATP, TNF-α, IL-1β, and IL-6, were measured using specific ELISA kits.

S1.7.3. Immunofluorescence staining of wound issue

Subsequent to the euthanasia of mice on days 6 and 11 following the establishment of the mouse model, CD31, CD68, and α-SMA assays were performed. All stained sections were observed and imaged using fluorescence microscopy, with full-field scanning performed and quantitative image analysis conducted using ImageJ 5.0 software.

S2. Supporting figures

Table S1. Compositional details of CPU, PU–Fe, and CPFSD–series composite dressings

Samples	PEG-IPDI prepolymer (wt%)	SD (Dopamine-grafted sodium alginate) (wt%)	Ferrocene (Fc) (wt%)	Curcumin (Cur) (wt%)
PU–Fe	98.5	0	1.0	0.5
CPU	98.5	0	0	1.5
CPFSD1	88.5	10.0	1.0	0.5
CPFSD2	78.5	20.0	1.0	0.5
CPFSD3	68.5	30.0	1.0	0.5
CPFSD4	58.5	40.0	1.0	0.5

All values are given in weight percent (wt%). SD, dopamine-grafted sodium alginate; PEG-IPDI, polyethylene glycol-isophorone diisocyanate.

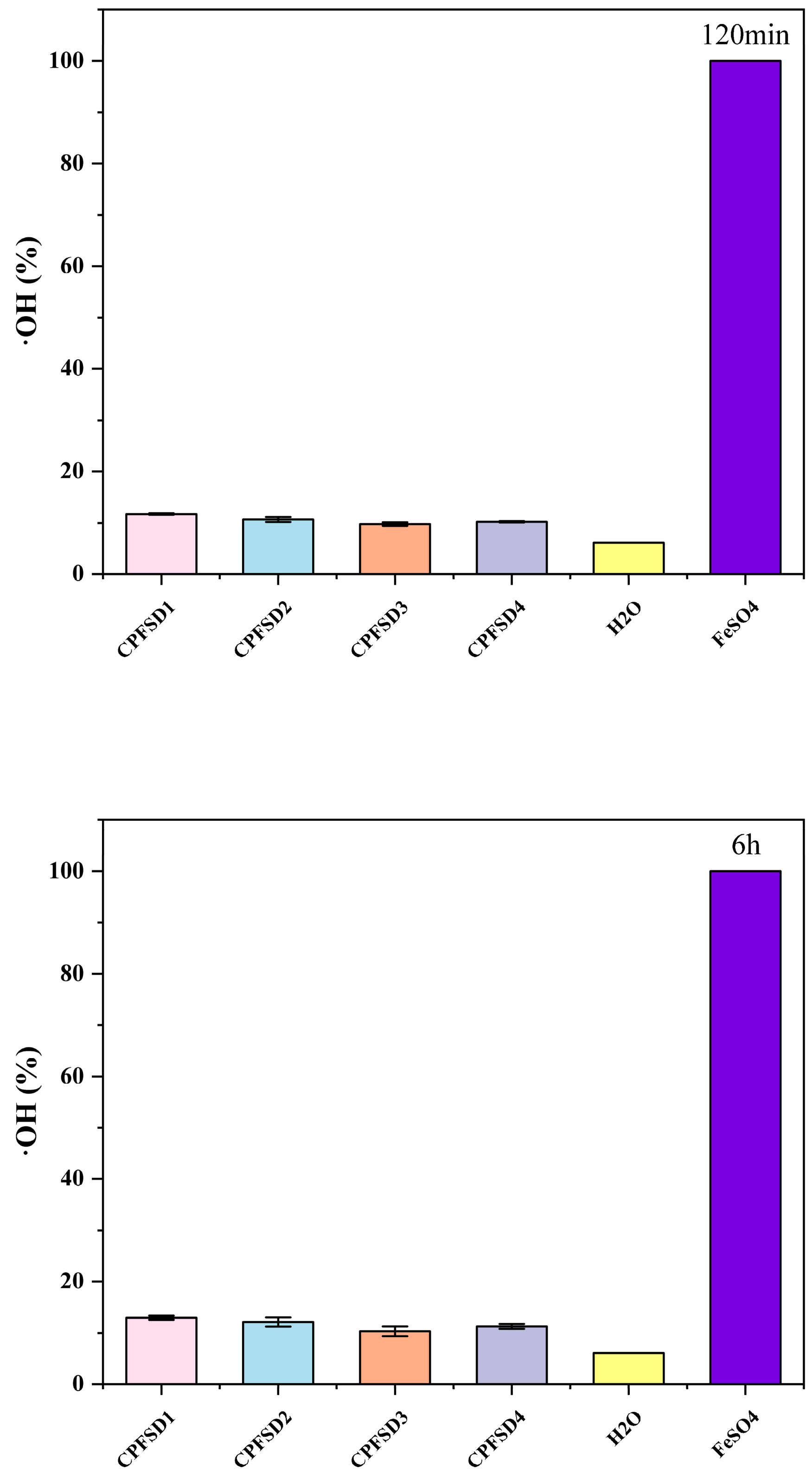

Figure S1. Promoting effects of water, CPFSD, and $FeSO_4$ on hydroxyl radicals in a short time

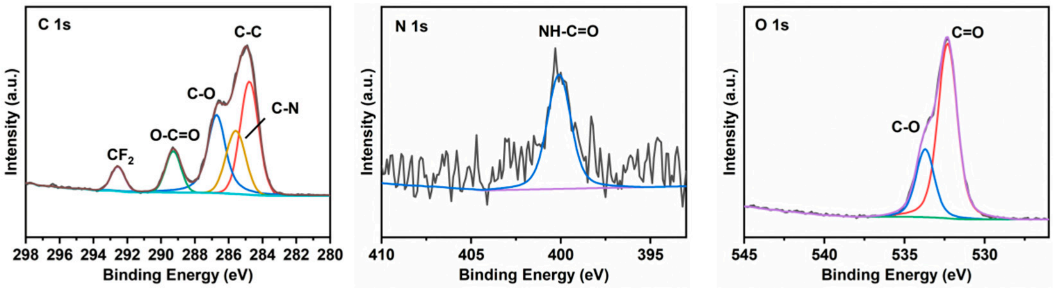

Figure S2. XPS of CPFSD

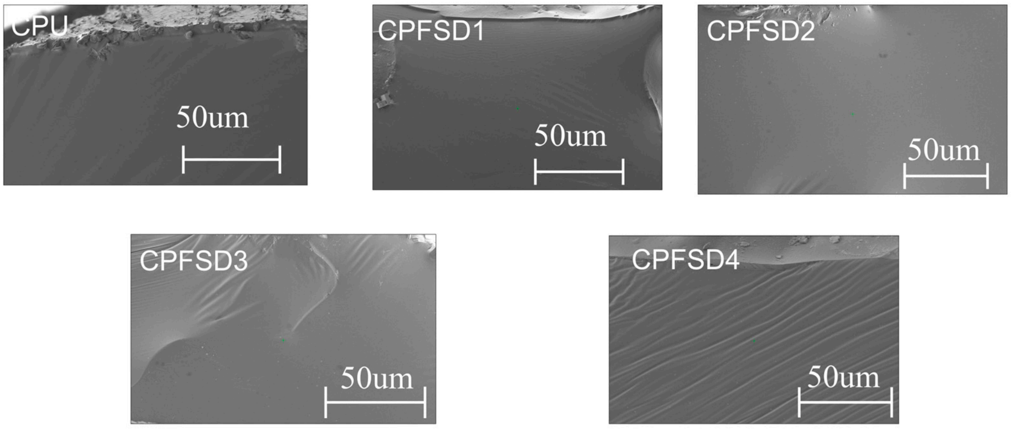

Figure S3. SEM images of CPU, CPFSD1, CPFSD2, CPFSD3, and CPFSD4

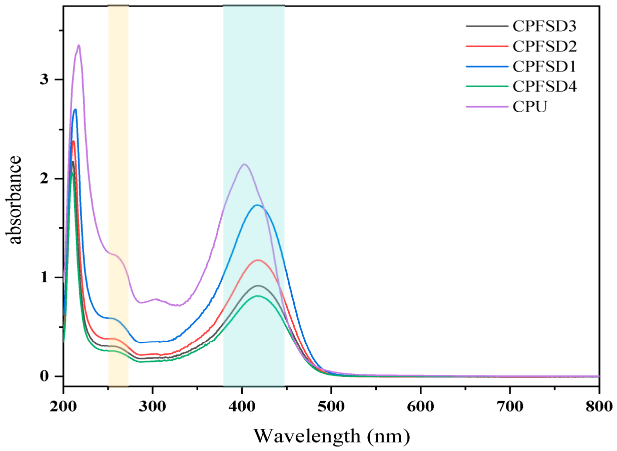

Figure S4. UV-Vis of CPFSD

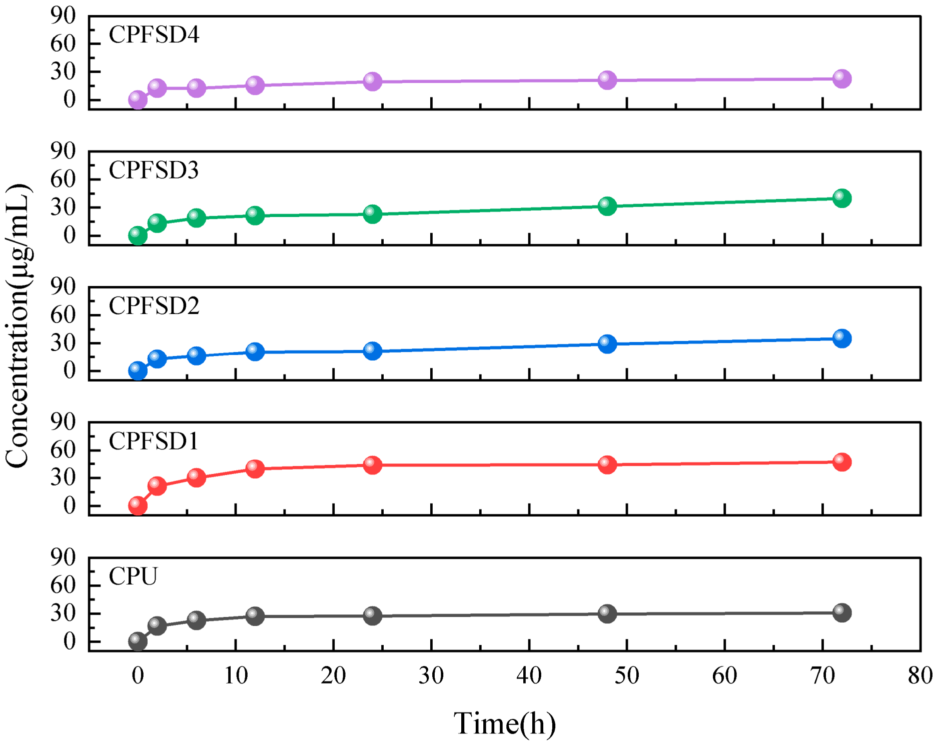

Figure S5. Cumulative curcumin release profiles. (pH=6.5)

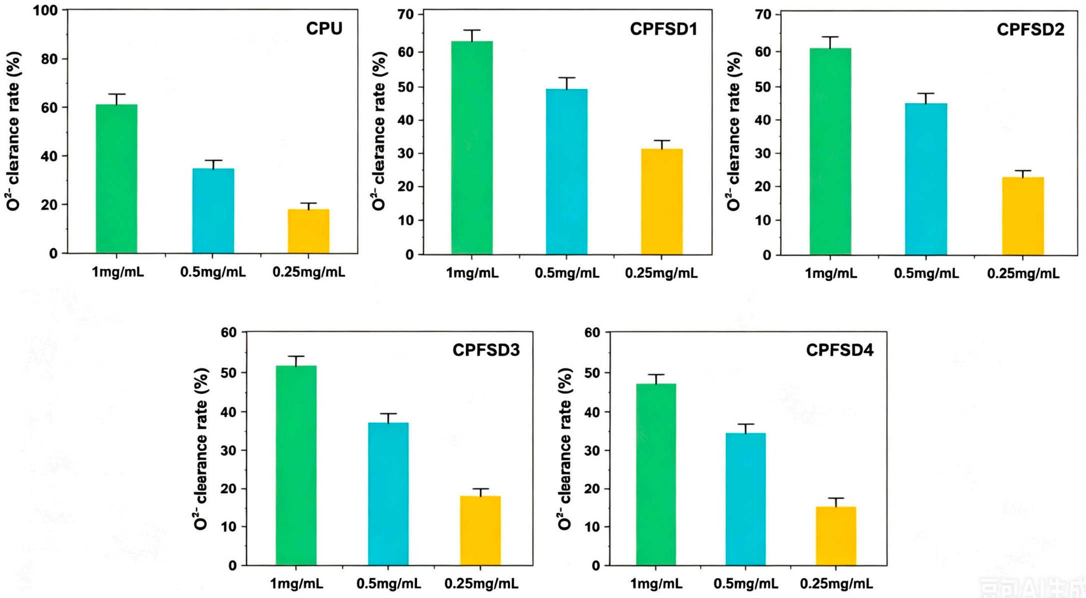

Figure S6. Effects of CPU, CPFSD1, CPFSD2, CPFSD3, and CPFSD4 dressings on the elimination of $O_2•^-$ at different concentrations.

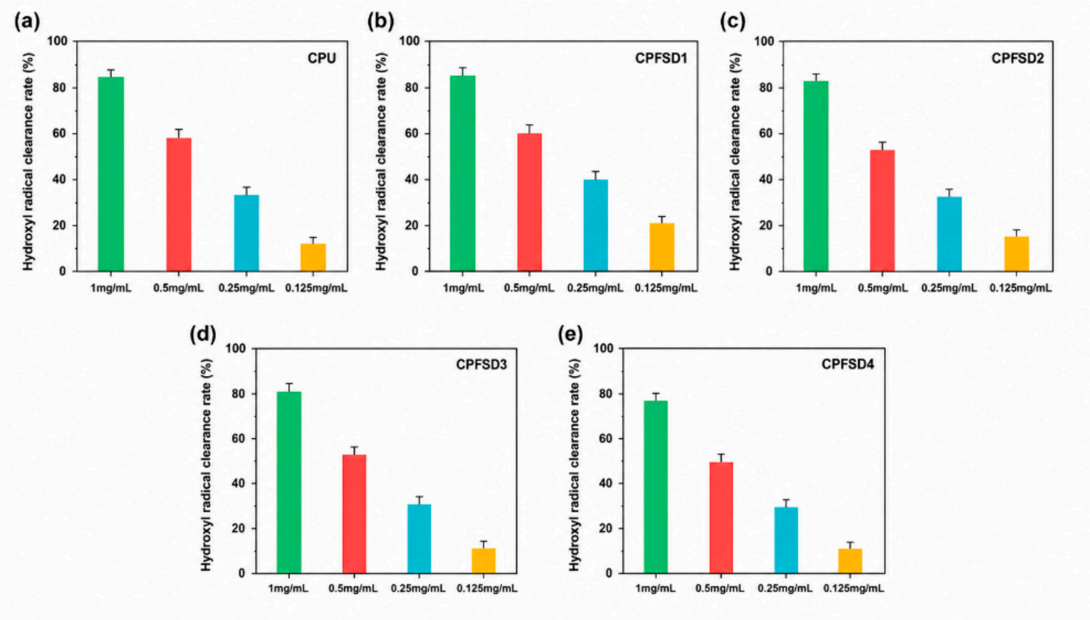

Figure S7. Effects of CPU, CPFSD1, CPFSD2, CPFSD3, and CPFSD4 dressings on the elimination of hydroxyl radicals (•OH) at different concentrations.

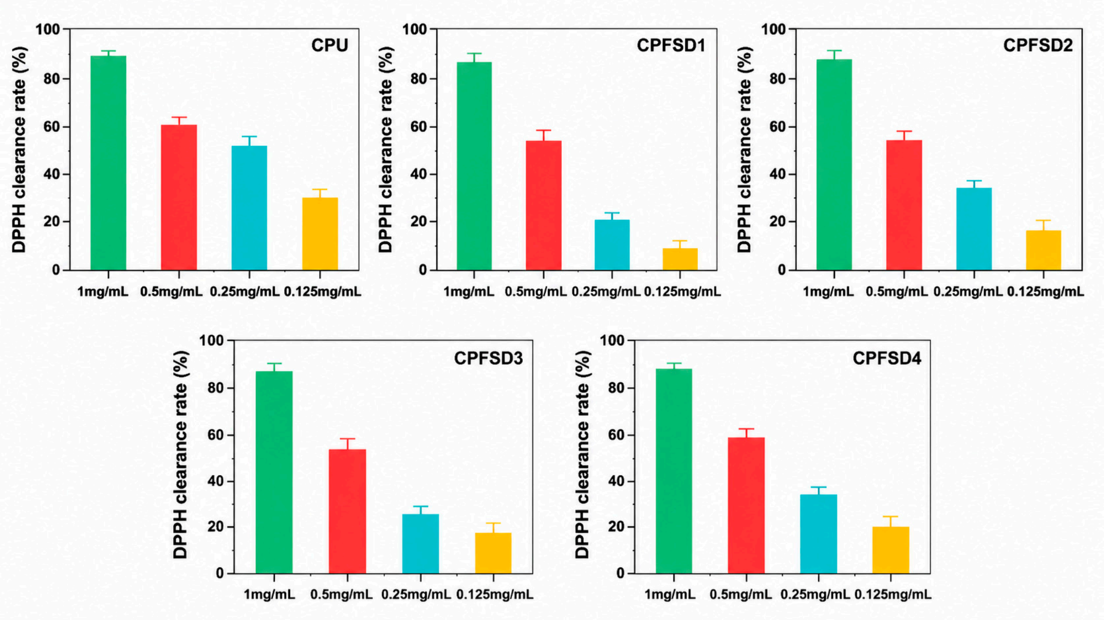

Figure S8. Effects of CPU, CPFSD1, CPFSD2, CPFSD3, and CPFSD4 dressings on the elimination of DPPH• at different concentrations.

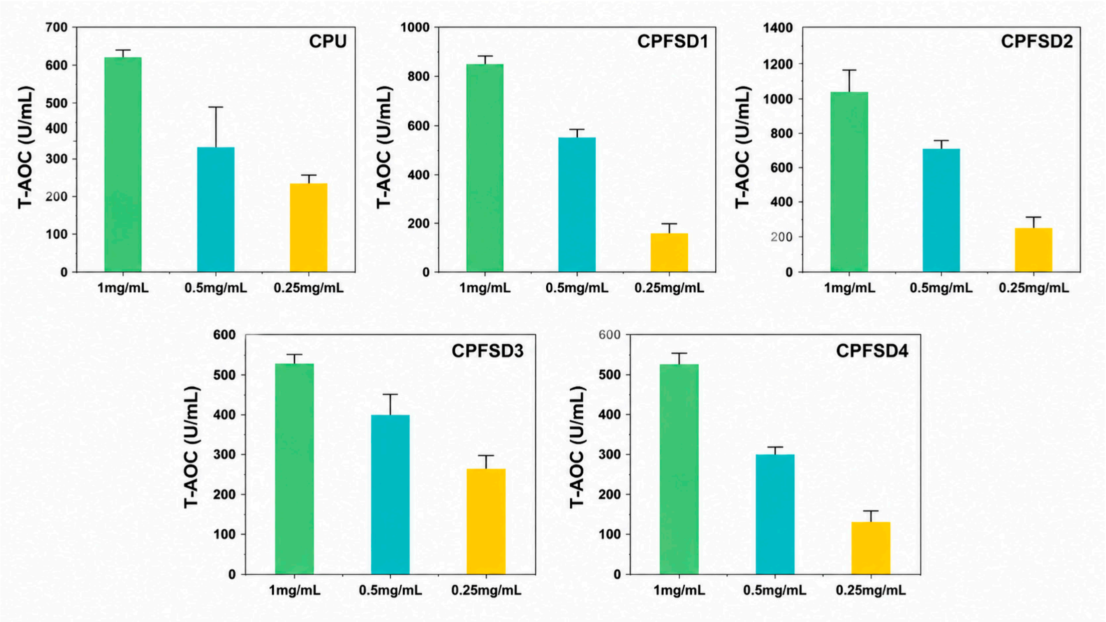

Figure S9. Effects of CPU, CPFSD1, CPFSD2, CPFSD3, and CPFSD4 dressings on the elimination of total antioxidant capacity (T-AOC) at different concentrations.

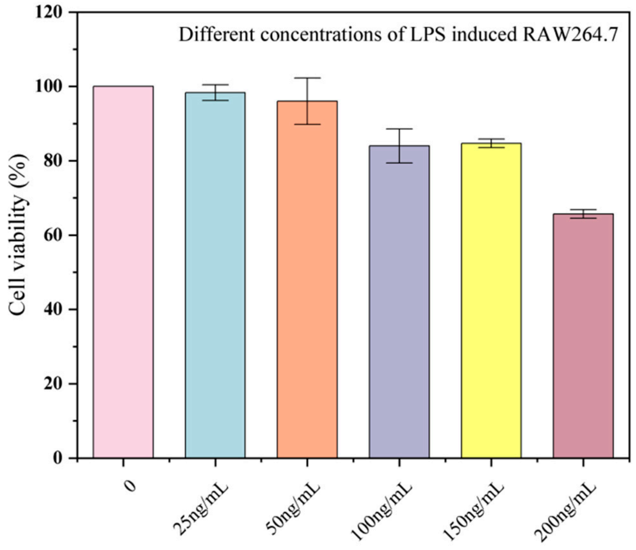

Figure S10. Different concentrations of LPS-induced RAW264.7

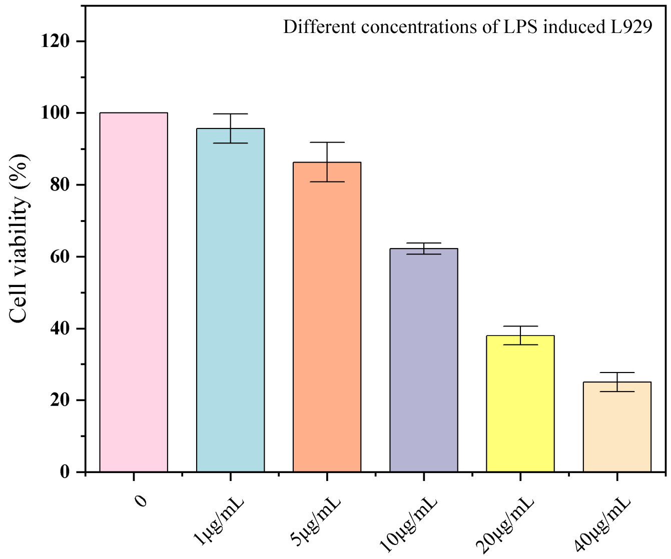

Figure S11. Different concentrations of LPS-induced L929

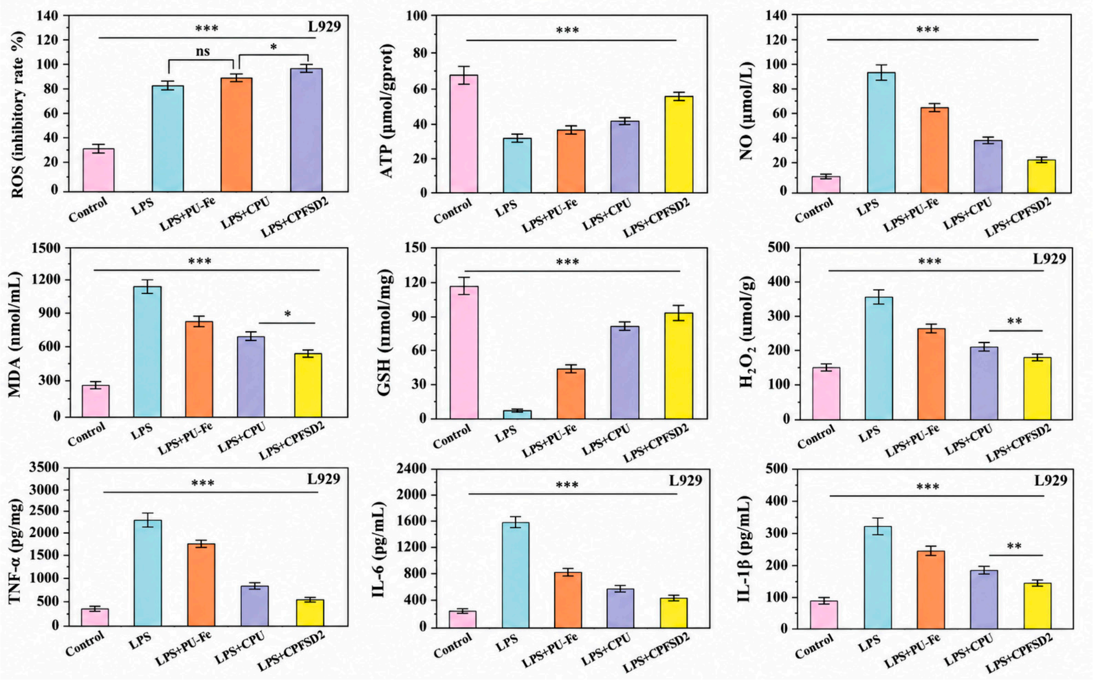

Figure S12. ROS-scavenging capacity of L929 cells under CPFSD dressing treatment

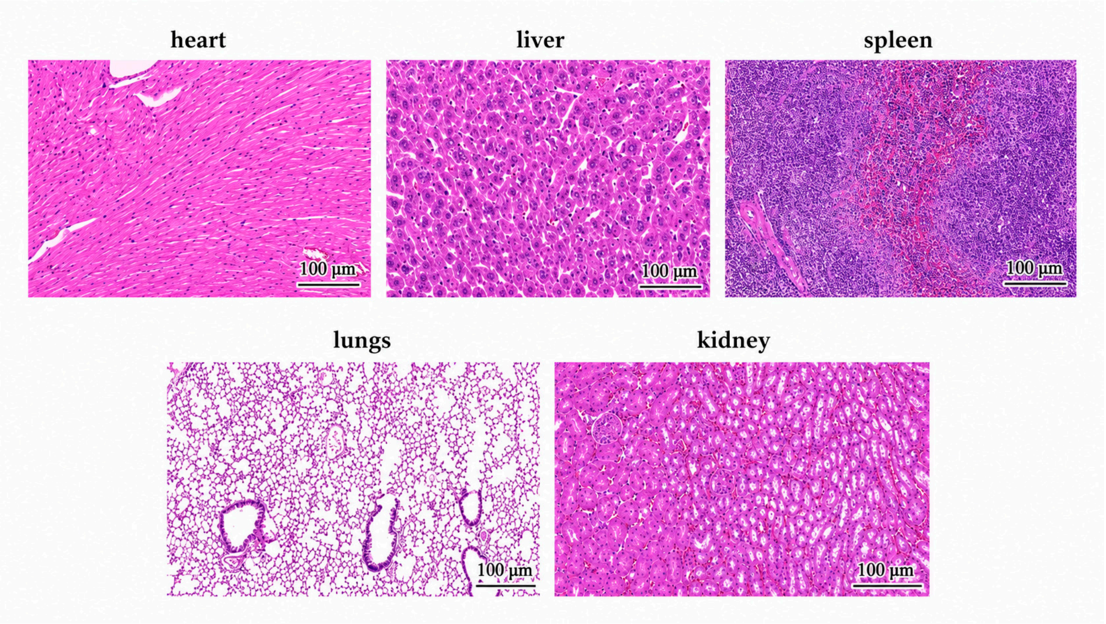

Figure S13. HE staining of heart, liver, spleen, lung, and kidney in mice with CPFSD2 dressing.
